# Proteogenic Dipeptides Are Characterized by Diel Fluctuations and Target of Rapamycin Complex-Signaling Dependency in the Model Plant *Arabidopsis thaliana*

**DOI:** 10.3389/fpls.2021.758933

**Published:** 2021-12-22

**Authors:** Maria Juliana Calderan-Rodrigues, Marcin Luzarowski, Carolina Cassano Monte-Bello, Romina I. Minen, Boris M. Zühlke, Zoran Nikoloski, Aleksandra Skirycz, Camila Caldana

**Affiliations:** ^1^Max Planck Institute of Molecular Plant Physiology, Potsdam, Germany; ^2^Boyce Thompson Institute, Ithaca, NY, United States; ^3^Institute for Biochemistry and Biology, University of Potsdam, Potsdam, Germany

**Keywords:** dipeptide, diel cycle, metabolism, TOR signaling, protein-metabolite interactions, carbon limitation, amino acid

## Abstract

As autotrophic organisms, plants capture light energy to convert carbon dioxide into ATP, nicotinamide adenine dinucleotide phosphate (NADPH), and sugars, which are essential for the biosynthesis of building blocks, storage, and growth. At night, metabolism and growth can be sustained by mobilizing carbon (C) reserves. In response to changing environmental conditions, such as light-dark cycles, the small-molecule regulation of enzymatic activities is critical for reprogramming cellular metabolism. We have recently demonstrated that proteogenic dipeptides, protein degradation products, act as metabolic switches at the interface of proteostasis and central metabolism in both plants and yeast. Dipeptides accumulate in response to the environmental changes and act *via* direct binding and regulation of critical enzymatic activities, enabling C flux distribution. Here, we provide evidence pointing to the involvement of dipeptides in the metabolic rewiring characteristics for the day-night cycle in plants. Specifically, we measured the abundance of 13 amino acids and 179 dipeptides over short- (SD) and long-day (LD) diel cycles, each with different light intensities. Of the measured dipeptides, 38 and eight were characterized by day-night oscillation in SD and LD, respectively, reaching maximum accumulation at the end of the day and then gradually falling in the night. Not only the number of dipeptides, but also the amplitude of the oscillation was higher in SD compared with LD conditions. Notably, rhythmic dipeptides were enriched in the glucogenic amino acids that can be converted into glucose. Considering the known role of Target of Rapamycin (TOR) signaling in regulating both autophagy and metabolism, we subsequently investigated whether diurnal fluctuations of dipeptides levels are dependent on the TOR Complex (TORC). The *Raptor1b* mutant (*raptor1b*), known for the substantial reduction of TOR kinase activity, was characterized by the augmented accumulation of dipeptides, which is especially pronounced under LD conditions. We were particularly intrigued by the group of 16 dipeptides, which, based on their oscillation under SD conditions and accumulation in *raptor1b*, can be associated with limited C availability or photoperiod. By mining existing protein-metabolite interaction data, we delineated putative protein interactors for a representative dipeptide Pro-Gln. The obtained list included enzymes of C and amino acid metabolism, which are also linked to the TORC-mediated metabolic network. Based on the obtained results, we speculate that the diurnal accumulation of dipeptides contributes to its metabolic adaptation in response to changes in C availability. We hypothesize that dipeptides would act as alternative respiratory substrates and by directly modulating the activity of the focal enzymes.

## Introduction

In a wide range of organisms, photoperiod length influences several vital events such as growth rate, reproduction, disease progression, and migration. The coordination of these internal events with the predictable photoperiod changes can optimize the use of resources, such as food availability and environmental conditions (Dardente et al., 2014). In plants, the length of light and night periods affect the following: biomass accumulation and growth (Nozue et al., 2007; Khoeyi et al., 2012; Doust, 2017), flowering (Garner and Allard, 1920; Yanovsky and Kay, 2002; Salazar et al., 2009), and cell wall composition and C allocation (Sulpice et al., 2014; Mengin et al., 2017; Alves et al., 2019). The adaptation to variation in light exposure is a competitive asset that allows plants to be tuned with foreseeable environmental changes following seasonal transitions. Moreover, plant development under different photoperiods establishes distinct internal dynamics by adjusting the metabolism to available sunlight (Seaton et al., 2018). This is particularly relevant for plants from high latitudes, as the growing season is getting longer due to climate changes, and thus, adaptation to the dynamic environment can be crucial in sustaining productivity [reviewed by Piao et al. (2019)].

Plants rely on the light supply to capture energy to convert carbon dioxide into ATP, nicotinamide adenine dinucleotide phosphate (NADPH), and sugars, which are essential for the biosynthesis of building blocks, cell proliferation, biomass accumulation, and reproductive fitness. In long photoperiods (LD) (16 h light/8 h dark), Arabidopsis transcript and protein levels match accelerated growth and have a faster transition to flowering, in comparison to short days (SD) (8 h light/16 h dark) (Baerenfaller et al., 2015). At night, plant growth is supplied by C skeletons released from the mobilization of transient starch that is synthesized during the light period (Smith and Stitt, 2007). Reasonably, the rates of C allocation into starch during the day are faster and its mobilization during the night is slower in SD condition, allowing this polysaccharide to last until the next light period (Sulpice et al., 2014; Mengin et al., 2017). The diel pattern of starch turnover optimizes growth under C limitation, in which, almost all of these compounds accumulated during the day were used to supply growth while avoiding C starvation, protein catabolism, and growth impairment caused by a premature C exhaustion at night (Moraes et al., 2019 and references therein). Starch accumulation pattern is more dependent on the duration of the light period rather than light intensity since shorter periods of light allocate a higher proportion of photosynthate into starch, whereas when irradiance fluctuated, this change was milder. The higher rate of starch accumulation in SD to sustain metabolism and growth at the longer nights leaves less C available for growth in the light period (Mengin et al., 2017), characterizing this photoperiod to display a smaller proportion of C that is available for growth compared with LD. Thus, when we mention C limitation or C restriction throughout the text related to our experiments, they are considered in terms of C mobilization to fuel growth rather than the photosynthetic rate. Both the decreased extent of diurnal turnover of C reserves and the transcriptional daytime changes in LD suggested that this photoperiod does not need tight energy management as SD (Baerenfaller et al., 2015). In addition to starch, autophagy also contributes in generating energy during longer night periods of SD under diurnal low light intensity, thus providing an alternative energy supply source as amino acids from protein catabolism (Izumi et al., 2013). Accordingly, transcripts and metabolic data related to autophagy increased during the dark even when the photoperiod comprised 14 h light, and had further augmented in the extended night (Usadel et al., 2008). Moreover, under conditions of starvation, like the one faced by the starchless *phosphoglucomutase* mutant (*pgm*) grown under SD, the rates of protein degradation increased rapidly from 4 h night onward, establishing a connection between C availability and protein degradation (Ishihara et al., 2015). Further, small Arabidopsis accessions cultivated in SD had presented higher ribosome synthesis while large accessions showed evidence of even protein degradation at night, indicating that the C-efficiency of growth would be decreased due to a higher energy cost in small accessions (Ishihara et al., 2017).

The “Target of Rapamycin” (TOR) kinase is an evolutionarily conserved key component in the network-regulating energy sensing into growth-mediated processes. The balance control between anabolism (e.g., biosynthesis of protein, lipids, and nucleotides) and catabolism (e.g., autophagy) in response to environmental cues are among the main functions of this pathway (Liu and Sabatini, 2020). The TOR complex (TORC) controls a plethora of metabolic pathways integrating energy status, like C and nitrogen (N) balance into biosynthetic growth in photosynthetic organisms (Caldana et al., 2013, 2019; Dobrenel et al., 2016; Jüppner et al., 2018; Monte-Bello et al., 2018; Mubeen et al., 2019; da Silva et al., 2021). TOR stimulates cell proliferation through light and its derived signals such as sugars and hormones (Pfeiffer et al., 2016; Li et al., 2017; Mohammed et al., 2018). As in other organisms, plant TORC is an autophagy repressor by inhibiting AuTophaGy (ATG) genes (Liu and Bassham, 2010) and possibly binding to the autophagy initiators ATG1-ATG13 (Van Leene et al., 2019). When purine levels are reduced, TOR activity is decreased into Arabidopsis T2 family endoribonuclease RNS2 (*rns2-2*) mutant, which in turn activates autophagy, suggesting that TOR mediates this catabolic process by responding to the levels of nucleotides to restore homeostasis (Kazibwe et al., 2020). As a consequence, the autophagy-induced recycling of nutrients can reactivate the TOR pathway, while nucleotides promote TOR activity (Busche et al., 2021).

In most eukaryotes, the TOR kinase is assembled into two distinct protein complexes, known as TORC1 and TORC2, which shared the same catalytic subunit TOR kinase and the regulatory subunit Lethal with Sec Thirteen8 (LST8). Their precise substrate recruitment and the physiological and biochemical distinction are mainly attributed to the regulatory subunits, the Regulatory-associated protein of TOR (RAPTOR) and Rapamycin-insensitive companion of mammalian target of rapamycin (RICTOR) in TORC1 and TORC2, respectively. In mammals, RAPTOR was identified as a mediator of the mTOR activity *in vivo* (Hara et al., 2002), then forming a nutrient-sensitive complex. Under poor nutrient conditions, RAPTOR and mTOR tight interaction destabilizes the complex and leads to reduced kinase activity (Kim et al., 2002). In plants, only TORC1 components have been so far identified (Menand et al., 2002; Mahfouz et al., 2006; Agredano-Moreno et al., 2007; Maegawa et al., 2015). However, the lack of viability or lethality of *tor* mutants (Menand et al., 2002; Ren et al., 2011) hampered research on the TOR pathway-mediated regulation in plants. To better understand TOR roles in plant development, the *raptor1b* T-DNA KO lines that displayed a strong reduction of TOR kinase activity, which were determined by assaying the ribosomal protein kinase 6 (S6K) activity (Salem et al., 2018), have been previously characterized. As other mutants from the complex (Moreau et al., 2012), *raptor1b* plants presented impaired development under LD photoperiod, such as massive changes in central C and N metabolism, increased levels of starch, free amino acids, and induced autophagy (Salem et al., 2017, 2018).

The small-molecule regulation of enzymatic activities is critical for the reprogramming of cellular metabolism in response to the changing environments across life kingdoms. Amino acids were proven to be activators of the TOR pathway-mediated impact on respiratory levels at night in plants (O’Leary et al., 2020), aside from restoring TOR activity under nitrogen starvation (Liu et al., 2021). Interestingly, the feeding of leukemia stem cells with specific dipeptides affects mTOR activity and nutrient signaling (Naka et al., 2015). Moreover, cyclodipeptides released by bacteria activated the TOR pathway and increased growth in plants (Corona-Sánchez et al., 2019; González-López et al., 2021). Recent system-wide characterization of the protein-protein-metabolite complexes in the model plant *Arabidopsis thaliana* provided evidence supporting the role of proteinogenic dipeptides in the regulation of enzymatic activities and C flux distribution (Veyel et al., 2017, 2018). For instance, an acidic dipeptide Tyr-Asp inhibits the activity of a key glycolytic enzyme glyceraldehyde 3-phosphate dehydrogenase, and as consequence, redirects glycolytic triose-phosphates toward the pentose phosphate pathway (PPP) and NADPH production (Moreno et al., 2021). Importantly, Tyr-Asp supplementation improved tobacco and Arabidopsis growth performance measured under oxidative stress conditions. Consistent with their role in metabolic adaptation to stress, acidic dipeptides, such as Tyr-Asp, accumulated in response to heat, dark, and microbial infection (Strehmel et al., 2017), and this observed accumulation is autophagy-dependent (Thirumalaikumar et al., 2020). Moreover, Glyceraldehyde 3-phosphate dehydrogenase (GAPDH) is not the sole glycolytic enzyme affected by dipeptides. For example, the inhibition of Arabidopsis phosphoenolpyruvate carboxykinase activity by the group of branched-chain amino acid (BCAA) containing dipeptides, but not by Tyr-Asp, points to a multisite regulation of glycolytic/gluconeogenic pathway by dipeptides. Proteolysis is the main source of dipeptides biogenesis, and autophagy has been addressed as the mechanism through which dipeptides are generated in plants under heat stress (Thirumalaikumar et al., 2020).

The emerging role of dipeptides as important players in shifting C metabolism has prompted us to investigate whether changes in photoperiod, light intensity, and thus C supply, would affect the accumulation of these signaling molecules along the diel cycle in *A. thaliana*. We found that a higher number of dipeptides have their levels oscillating under SD (8 h light/16 h dark, 20°C/16°C, 180 μmol m^–2^ s^–1^) than LD (16 h light, 8 h dark, 20°C/16°C, 120 μmol m^–2^ s^–1^). To get further insights into the role of these dipeptides in rewiring metabolism under SD, we took the advantage of the mutant involved in energy sensing, *raptor1b*, which presents a photoperiod-condition phenotype. The greater the C availability the stronger is the metabolic phenotype (Deprost et al., 2007; Moreau et al., 2012; Salem et al., 2018), at the same time that de-repress autophagy machinery (Salem et al., 2018). As expected, when compared with Col-0, *raptor1b* presents a larger number of dipeptides with altered levels under LD. By focusing on dipeptides that present an oscillating pattern along the diel cycle in Col-0 only under SD conditions, and always enhanced levels in *raptor1b*, we have identified a group of 16 dipeptides that can be possibly associated with C-restricted supply. By mining existing protein-metabolite interaction data (Zühlke et al., 2021), we delineated putative protein interactors for a representative dipeptide Pro-Gln. The obtained list included enzymes of C and amino acid metabolism, which are also linked to the TORC-mediated metabolic network. If taken together, our data strengthen published findings (Thirumalaikumar et al., 2020; Luzarowski et al., 2021; Moreno et al., 2021), supporting the role of dipeptides in rewiring metabolism under nutrient restrictions, which needs to be further explored.

## Materials and Methods

### Plant Material and Growth Conditions

**FIGURE 1:**
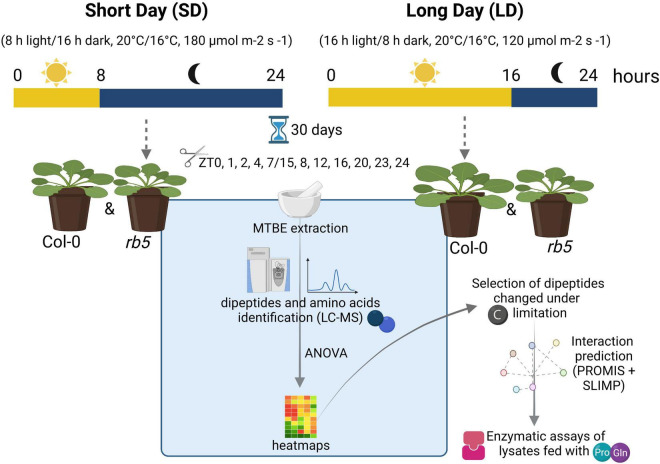
Overview of the experimental design. *rb5* is the abbreviated form for *raptor1b*. Created with BioRender.com.

Plants of *Arabidopsis thaliana Columbia-0* (Col-0) and *rb10* (*raptor1b* T-DNA KO line; SALK_101990) were grown in soil in a controlled environment chamber in SD (8 h light/16 h dark, 20°C/16°C, 180 μmol m^–2^ s^–1^) and LD (16 h light, 8 h dark, 20°C/16°C, 120 μmol m^–2^ s^–1^) photoperiods under a relative humidity of 75% (see Figure 1 for details on experimental design). After 30 days of growth, whole rosettes were harvested every 4 h from Zeitgeber time (ZT) 0 and additional time points were added during the light- dark transitions (ZT 1, 2, 23, and 1 h before the start of the dark phase, i.e., 7 or 15, depending on the photoperiod) and were immediately frozen in liquid nitrogen. Frozen plant tissue was ground into a fine powder and stored at −80°C until use. Five biological replicates consisted of pools were composed of 15 or 5 plants harvested for SD and LD, respectively.

For the enzymatic measurements (see section “Enzymatic Activity Assay”), *A. thaliana* Col-0 plants were grown under a 12 h light/12 h dark diel cycle in a chamber at 23°C and with a light intensity of 100 μmol m^2^ s^–1^. Rosette leaves from 4-week-old plants were harvested 2 h into light or 2 h into dark, and rapidly frozen with liquid nitrogen. Plant material was ground into a fine powder and stored at −80°C until use.

### Metabolite Analysis

Metabolites were extracted from 50 mg of fresh rosette material following the method described by Giavalisco et al. (2011), with modifications. A volume of 1 mL of the precooled methyl-tert-butyl-ether (MTBE) extraction mixture (−20°C) was added to the homogenized tissues. Subsequently, the tubes were then homogenized using vortex and incubated in a shaker (100 rpm, 10 min, 4°C), followed by 10 min of sonication. This extraction allows the separation between the polar and apolar phases. For this separation, 500 μL of the methanol: water (1:3/v:v) mixture was added to each tube and the samples were mixed in the vortex (5 min). The samples were centrifuged (20,000 *g*, 10 min, 4°C), harvested, concentrated (SpeedVac), and stored at −80°C until liquid chromatography-mass spectrometry (LC-MS) analysis. Two volumes of the polar phase were used for liquid chromatography (LC) analyses.

The dried aqueous phase was solubilized in 200 μL of high-performance LC (HPLC)-grade water and sonicated for 5 min using an ultrasonication bath. Samples were centrifuged for 10 min at 20,800 *g*, RT. The supernatant was transferred to the UPLC glass vial. A 3 μl of polar metabolite extract was separated using an ultra-performance LC (UPLC) equipped with an HSS T3 C18 reversed-phase column at a 400 μl/min flow rate. Mass spectra were acquired using an Exactive mass spectrometer in positive ionization mode (Giavalisco et al., 2011). Mobile phase solutions were prepared as follows: buffer A (0.1% formic acid in H2O) and buffer B (0.1% formic acid in ACN). The following gradients were used for metabolite separation: 1 min 1% LC-MS mobile phase buffer B, 11 min linear gradient from 1 to 40% buffer B, 13 min linear gradient from 40% to 70% buffer B, then 15 min linear gradient from 70 to 99% buffer B, and held a 99% buffer B concentration until 16 min. Starting from 17 min, a linear gradient from 99 to 1% buffer B was used. The column was re-equilibrated for 3 min with 1% buffer B before the next measurement was performed. Mass spectra were acquired using the following settings: mass range from 100 to 1,500 *m/z*, resolution set to 25,000, loading time restricted to 100 ms, AGC target set to 1e^6^, capillary voltage to 3 kV with a sheath gas flow, and auxiliary gas value of 60 and 20, respectively. The capillary temperature was set to 250°C and skimmer voltage to 25 V.

#### Data Processing

Data were processed using Expressionist Refiner MS 14.0.5 (Genedata AG, Basel, Switzerland) as previously described (Veyel et al., 2017; Sokolowska et al., 2019). Minor changes were applied to the workflow: chromatogram alignment (RT search interval 0.5 min) and peak detection (minimum peak size 0.03 min, gap/peak ratio 50%, smoothing window 5 points, center computation by intensity-weighted method with the threshold at 70%, boundary determination using inflection points). Detailed instructions for using the software can be found in prior work (Sokolowska et al., 2019).

The processing of 95 and 105 samples, collected from both SD and LD conditions, resulted in the detection of 135,294 and 245,041 peaks (mass features), respectively. Subsequently, mass features were filtered for these with intensity above 10,000 presents in at least 32 and 29% of the samples at SD and LD photoperiods, respectively. Intensities of remaining mass features were medianly normalized and annotated using the in-house reference compound library as described below.

#### Annotation

Mass features described by RT and m/z were matched to in-house libraries of authentic reference compounds, allowing a 0.005 Da mass and dynamic retention time deviation (maximum 0.2 min). This enabled the identification of 130 and 168 metabolites in SD and LD conditions, respectively (Supplementary Datasets 1–4). In both Supplementary Datasets 1, 3, there were 110 common metabolites detected.

#### Generation of Heatmaps

**FIGURE 2:**
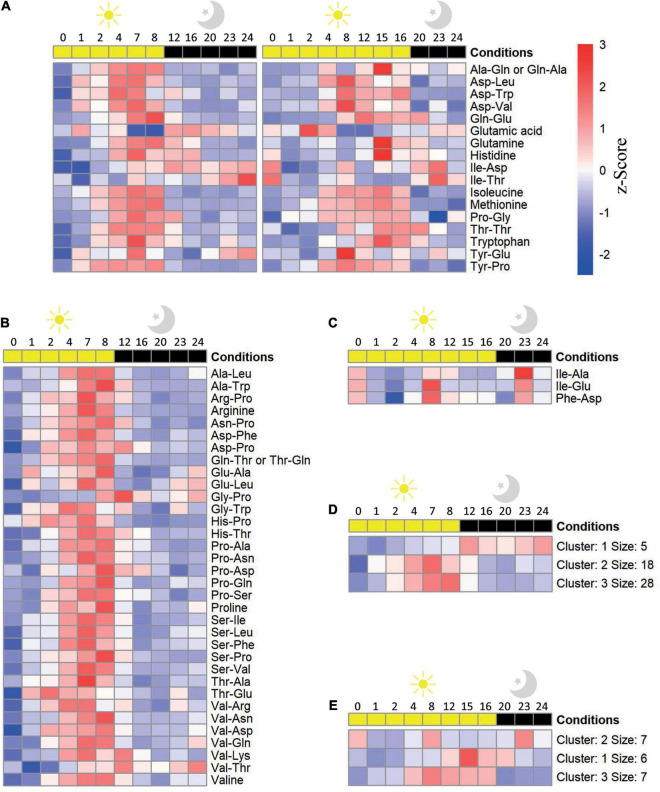
Oscillation of amino acid and dipeptide levels in *Arabidopsis thaliana* Col-0 plants throughout a diel cycle (24 h). Dipeptides and amino acid levels that were significantly (adjusted *P* value ≤ 0.05) affected throughout a diel cycle at **(A)** short- (SD) and long day (LD), **(B)** solely at SD, and **(C)** solely at LD conditions are illustrated as a heatmap. Metabolites were ordered alphabetically. Shown is scaled abundance. K-means clustering of the metabolites levels at **(D)** SD and **(E)** LD was performed with *k* = 3. *n* biological replicates = 3–5.

Intensities of 110 metabolites found in SD and LD conditions were log_2_-transformed and the missing values (0) were replaced by NA. Metabolites whose intensities were significantly affected during the experiment were determined using one-way ANOVA, followed by Bonferroni’s correction for multiple comparisons (adjusted *P* < 0.05) (Supplementary Tables 1, 2). Subsequently, the mean intensity of three to five biological replicates was scaled using the “scale” function in R. Scaled, the mean intensity of metabolites significantly affected during the experiment in wild type (WT), and *raptor1b* mutant were shown in Figure 2 and in Supplementary Figures 1–3.

**FIGURE 3:**
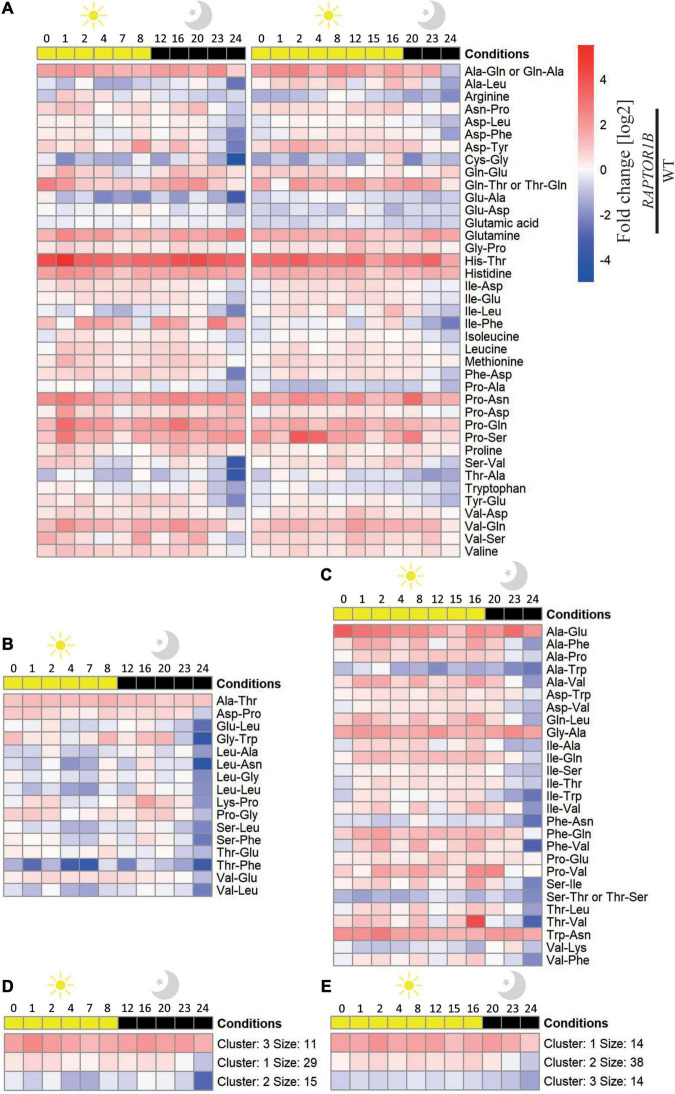
Fold change values of the oscillating amino acid and dipeptide levels in *A. thaliana raptor1b* over Col-0 plants throughout a diel cycle (24 h). Fold change [log_2_] of the dipeptides and amino acid levels significantly (adjusted *P*-value ≤ 0.05) affected throughout a diel cycle at **(A)** SD and LD, **(B)** solely at SD, and **(C)** solely at LD conditions are illustrated as a heatmap. Metabolites were ordered alphabetically. K-means clustering of the levels of the metabolites at **(D)** SD and **(E)** LD was performed with *k* = 3. *n* biological replicates = 3–5.

Two-way ANOVA, followed by Bonferroni’s correction for multiple comparisons (adjusted *P* < 0.05) were used to determine metabolites that are significantly affected by time, gene deletion, or both factors (Supplementary Table 3). Next, the mean intensity of three to five biological replicates was calculated for each time point (Supplementary Tables 4, 5). Finally, intensity fold change was calculated between *raptor1b* and Col-0 plants (Figure 3 and Supplementary Figures 4A,B).

Heatmaps were generated using the “pheatmap” package. The R-packages used for data processing, analysis and visualization were mentioned in the GitHub repository^1^. The heatmaps are presented in two different ways: (i) (A) grouping common dipeptides, whose level was affected in both photoperiods, and the ones either affected solely in (B) SD or (C) LD condition alone (Figures 2, 3; Supplementary Figure 2); K-means clustering was performed with *k* = 3, and (ii) clustering the oscillating dipeptides by computing Euclidean distance between the rows. Clustering was performed using the complete method (Supplementary Figures 1A,B, 3, 4). Thus, the data presented in Figure 2 correlate with Supplementary Figure 1, Figure 3, and Supplementary Figure 4 are compatible, and Supplementary Figure 2 corresponds to Supplementary Figure 3.

#### Interactome Analysis

Pro-Gln interactors were retrieved from SLIMP (Zühlke et al., 2021). Protein-protein interactions were imported from STRING (Szklarczyk et al., 2016; Supplementary Table 6), and used to build the interaction network of the Pro-Gln interactors into Cytoscape (Shannon et al., 2003; Supplementary Table 7).

### Enzymatic Activity Assay

Soluble proteins were extracted from 20 mg of Arabidopsis material by addition of 10 mg (w/v) polyvinylpolypyrrolidone and 1 mL ice-cold extraction buffer, shaken vigorously and incubated 5 min in ice. The extraction buffer was composed of 50 mM HEPES/KOH, pH 7.5, 10 mM MgCl_2_, 1 mM EDTA, 1 mM EGTA, 1 mM benzamidine, 1 mM ε-aminocaproic acid, 0.25% (w/v) BSA, 10 μM leupeptin, 0.5 mM DTT, 0.1% (v/v) Triton X-100, 20% (v/v) glycerol, and 1 mM phenylmethylsulfonyl fluoride. The lysate was centrifuged for 10 min at 14,000 *g* and 4°C (Gibon et al., 2004).

The mitochondrial isocitrate dehydrogenase (ICDHP) activity was measured in conditions adapted from Sadka et al. (2000). The standard reaction media contained both 100 mM Tris–HCl pH 7.5, 10 mM MgCl_2_, 0.25 mM NADP^+^, and 2 mM D,L-isocitrate. Glyceraldehyde-3-phosphate dehydrogenase (GAPC), glucose-6-phosphate dehydrogenase (G6PDH), and 6-phosphogluconate dehydrogenase (6PGDH) were assayed as described by Rius et al. (2006), with minor modifications. The GAPC assay premix consisted of 50 mM Tricine-KOH pH 8.5, 4 mM NAD^+^, 1.2 mM fructose 1,6-bisphosphate (FBP), 10 mM sodium arsenate, and 1 U/ml aldolase from rabbit muscle (Sigma). G6PDH and 6PGDH assay mixture was composed by 100 mM Tris–HCl pH 8.0, 10 mM MgCl_2_, 0.5 mM EDTA, 5 mM DTT, 0.25 mM NADP^+^, and 1 mM G6P or 6PG, respectively. All measurements were performed in a final volume of 50 μl at 25°C under control (mock) conditions or with the addition of 100 μM Pro, Gln, or with the dipeptide Pro-Gln. One unit of enzyme activity (U) is defined as the amount of enzyme catalyzing the formation of 1 μmol of NAD(P)H per min under the above-described conditions.

## Results

### Amino Acid and Dipeptide Levels Oscillate Throughout a Diel Cycle

The accumulation of dipeptides in response to environmental cues and their roles as signals rewiring the metabolism (Liu and Christians, 1994; Naka et al., 2015; Strehmel et al., 2017; Doppler et al., 2019) prompted us to investigate whether the accumulation of dipeptides is dependent on the photoperiod. For this aim, we assessed the profile of dipeptides in *A. thaliana* plants cultivated for 30 days under SD and LD photoperiods, which are two contrasting conditions in terms of light and dark periods. In this analysis, we also included the profile of 13 amino acids to understand whether there is any correlation between the composition of dipeptide and the accumulation of the amino acids. To identify the pattern of the small molecules which correlates to the changes in the diel cycle, we performed a K-means clustering analysis (Figure 2).

A total of 11 dipeptides and six amino acids displayed an oscillation pattern along the diel cycle in both conditions, predominantly increasing during the day while decreasing at night. Majority of these compounds peak from the middle of the light period onward (ZT 4 and 8, in SD and LD, respectively) (Figure 2A), matching the augmented time of light exposure.

In LD condition, far less proteogenic dipeptides showed significant oscillation along the diel cycle (Figures 2A,C) and this trend was not related to the total amount of identified dipeptides in the two growth conditions (see “Materials and Methods” section). Regarding small molecules accumulation, three main clusters were identified: (i) with overall decreased levels along the diel cycle with two peaks of increase at the middle of the day and night (cluster 2); (ii) early (ZT4, cluster 3), and (iii) late (ZT8, cluster 1) accumulation in the light followed by a reduction in the dark (Figure 2E). Dipeptides from clusters 1 and 3 were mostly composed of non-aromatic amino acids containing linear aliphatic side chains, with exception of Val and Leu in the C-terminus. On the other hand, four out of the seven dipeptides from Cluster 2 present Ile at the N-terminus.

Reasonably, under the SD photoperiod, a higher number of dipeptides presented changes in their levels along the diel cycle in SD compared with LD (Figure 2). In the SD photoperiod, nine amino acids and 42 dipeptides had their levels significantly affected during the diel cycle considering the sum of molecules altered in both photoperiods and the ones solely oscillating under SD (Figures 2A,B). The vast majority of these compounds (38 dipeptides and 8 amino acids) (Figure 2D) enhanced their levels in response to light, reaching a maximum level at the end of the day and then gradually decreasing during the night. Such pattern was grouped into two clusters, 2 and 3, which mainly differ in the timing of the response. These dipeptides are mostly composed of the glucogenic amino acids Asp, Pro, Ser, and Val at the N-terminus, whereas at the C-terminus, only Pro was overrepresented. Despite being part of cluster 2, the Glu-containing dipeptides Glu-Ala, Glu-Leu, and Tyr-Glu displayed a discrete increase at night. Cluster 1 included only 4 dipeptides and Glu, presenting augmented levels predominantly at dark. Out of the 4 dipeptides from Cluster 1, only two, Gly-Pro and Val-Thr, were specific with SD condition.

A reasonable number of dipeptides from samples harvested at 0 and 24 h present differential levels in these time points (Figures 2, 3). Indeed, we expected similar behavior of the molecules within compatible time points following a 24 h cycle. To test whether the observed dipeptides accumulation follows the 24 h cycle, we compared the level of these molecules in these two time points. For about 68 to 80% of the molecules that show diurnal changes in the accumulation in either or both SD and LD conditions, the levels, even if not identical, are not significantly different (*P*-value > 005). It is important to note that due to the size of experiments, harvesting the material takes minutes, and hence time 0 h may not be exactly identical to the 24 h, which could contribute to a level of discrepancy. In the future, it will be interesting to follow dipeptide levels across multiple days to estimate the amplitude and frequency of the oscillations accurately.

### Mutation in the Substrate Recruiter of TOR Complex Increases Dipeptide Levels in LD Condition

To further explore the role of these dipeptides, we consequently performed a similar experiment using the mutant RAPTOR1B, *raptor1b*, which is the regulatory unit of the TORCI, known to sense energy and nutrient status to control protein synthesis and autophagy (Salem et al., 2018). TORC mutants have much more marked phenotypic changes under LD conditions (Deprost et al., 2007; Moreau et al., 2012; Salem et al., 2018), at the same time that de-repress autophagy machinery. Therefore, the induced autophagy characteristic of *raptor1b* under LD condition (Salem et al., 2018) places this mutant as a good candidate to validate the hypothesis that these dipeptide levels are dependent on autophagy to rewire the metabolism according to the diel cycle.

Similar to the Col-0, there was a higher number of dipeptides and amino acids oscillating in SD compared to LD in *raptor1b* (Supplementary Figures 2A–E, 3A,B). However, when the levels of these dipeptides were compared relative to the ones in Col-0, two main trends were observed. First, the oscillation pattern of these compounds was almost abolished along the diel cycle in both photoperiods, if the fold change was considered (Figures 3A–E). Second, there was a larger number of dipeptides presenting significant changes in *raptor1b* compared with Col-0 in LD (Figures 3A,C), supporting the stronger metabolic phenotype observed in *raptor1b* under this photoperiod (Salem et al., 2018). Interestingly, majority of these dipeptides were accumulated along the diel cycle in both photoperiods. However, the magnitude of these changes was, in general, more pronounced in longer photoperiods. Only 21 and 36% dipeptides with significant differences in Col-0 and *raptor1b* presented continuously reduced levels in LD and SD, respectively (Figure 3A). To identify candidates that would respond to limited C availability in an autophagy-dependent manner, we focused on the dipeptides presenting oscillating levels in Col-0 only under SD conditions, whereas in *raptor1b* the response should always be altered in relation to the WT (Figures 2B, 3A). This analysis pointed to alterations in the levels of the amino acids Arg, Pro, and Val and 16 dipeptides. Interestingly, seven dipeptides contained Pro (Gly-Pro, Pro-Ser, Asn-Pro, Pro-Ala, Pro-Asn, Pro-Asp, Pro-Gln), whereas four were composed by the BCAAs Val (Ser-Val, Val-Asp, Val-Gln) and Leu (Ala-Leu). Except for Gly-Pro, the levels of these compounds increase during the day and decrease at night in Col-0 under SD conditions (Figure 2B).

### Putative Pro-Gln Interactors Include Multiple Enzymes for the Central Carbon and Amino Acid Metabolism

By using PROMIS, a biochemical approach that relies on the co-fractionation-mass spectrometry (CF-MS) of proteins and associated small-molecule ligands in the cellular lysate (Veyel et al., 2017, 2018; Luzarowski et al., 2021), we demonstrated that dipeptides are present in the protein complexes. However, the main limitation of CF-MS is that every metabolite usually co-elutes with hundreds of different proteins. By combining PROMIS with orthogonal small-molecule centric approaches, such as affinity purification and thermal proteome profiling, we identified and verified protein targets for selected dipeptides. We became particularly intrigued by the protein-metabolite interactions between proteinogenic dipeptides and the enzymes of central C metabolism. The mechanistic characterization of the interaction between different dipeptides and proteins revealed that Tyr-Asp and BCAA-containing dipeptides displayed an inhibitory effect on distinct proteins, attesting the multisite regulation of C metabolism by different dipeptides (Moreno et al., 2021).

**FIGURE 4:**
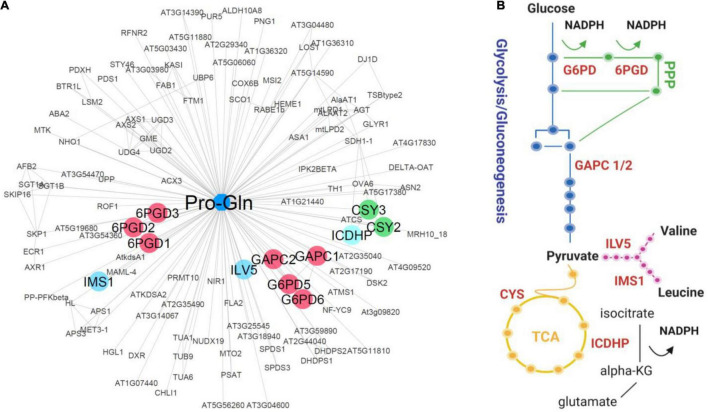
Pro-Gln/protein interaction network. **(A)** Pro-Gln interactors were predicted by SLIMP (Zühlke et al., 2021) with a score above 0.5. Protein-protein interactions were imported from STRING (Szklarczyk et al., 2016) based on the experimental and database evidence. Edge length is inversely proportional to either a STRING (PPI) or SLIMP (PMI) score. The network was created using Cytoscape (Shannon et al., 2003). Indicated proteins were selected based on their functions in C and amino acid metabolism. **(B)** Schematic representation of the metabolic pathways was created using Biorender.com.

To test whether the sixteen dipeptides delineated by our analysis (Figures 2B, 3A) are also involved in rewiring metabolism under C restricted conditions, we looked for the enzymes of central C and amino acid metabolism among their putative protein interactors. We first queried the STITCH (Kuhn et al., 2008) and BRENDA (Jeske et al., 2019) databases for known interactors for the 16 dipeptides, but we found none. As a result, we then exploited a recent set of predicted protein-metabolite interactions obtained by combining three different PROMIS experiments with supervised machine learning prediction of the interactors (SLIMP) (Zühlke et al., 2021). SLIMP helps to distinguish between true and coincidental interactors by looking for co-elution across the multiple separations. Out of the sixteen selected dipeptides, seven are involved in the predictions of protein-metabolite interactions by SLIMP, including one of the six Pro containing dipeptides, the Pro-Gln. The list of the predicted Pro-Gln interactors comprised 124 proteins, of which some were involved in the TOR pathway and critical metabolic enzymes (Figure 4A; Supplementary Tables 6, 7). We mined these 124 proteins to identify those involved in C metabolism. In addition to the GAPC 1 and 2, which we previously showed to be regulated by the dipeptide Tyr-Asp (Moreno et al., 2021), the list comprised two cytosolic isoforms of G6PDH, 5, and 6, and all the three Arabidopsis isoforms of 6PGDH (Figures 4A,B; Supplementary Table 6). Furthermore, the list of predicted Pro-Gln interactors contained enzymes related to the tricarboxylic acid cycle (TCA cycle) activity, as peroxisomal citrate synthases (CSY2 and CSY3) and ICDHP (Figure 4; Supplementary Table 6). Finally, the Pro-Gln interaction network is comprised of two enzymes of BCAA biosynthesis 2: isopropylmalate synthase (IMS1) and ketol-acid reductoisomerase (ILV5) (Supplementary Table 6). To follow-up on the interaction data, we measured enzymatic activity of the GAPC, G6PDH, 6PGDH, and ICDHP in the lysate prepared from the Arabidopsis plants grown under 12 h/12 h regime, harvested either 2 h into the light or 2 h into the dark and supplemented with either 100 μM Pro-Gln, Pro or Gln. In contrast to the previously published effect of Tyr-Asp on GAPC or Ala-Ile on PEPCK activity (Moreno et al., 2021), we measured no difference for the tested enzyme-ligand combinations (Supplementary Figures 5A–D). Thus, in our experimental conditions, Pro-Gln did not show a biologically relevant role in the regulation of the activity of these four enzymes associated with C metabolism.

## Discussion

The length of light and dark cycles in a day affects not only the rates of transcription (Baerenfaller et al., 2015) and translation (Sulpice et al., 2014) but also several metabolic processes (Seaton et al., 2018), including the diurnal turnover of C reserves, which ultimately impacted growth (Baerenfaller et al., 2015). Since SD and LD have an opposite duration of light and dark periods, we evaluated how these conditions would influence the collection of dipeptides in Arabidopsis.

Due to the light-dependent accumulation pattern of the 11 dipeptides in both photoperiods, their oscillation is not strictly related to the control of C-dependent metabolism. Most of the dipeptides with augmented levels at night were composed of glucogenic BCAAs at the N-terminus, in both photoperiods (Figure 2). Glucogenic and ketogenic amino acids can be distinguished by their ability or inability to be used to generate glucose. The dipeptides composed of glucogenic amino acids could be mobilized and generate the C skeletons to produce pyruvate and then glucose through gluconeogenesis (Eastmond et al., 2015). As the duration of the night dramatically influences C availability for growth (Sulpice et al., 2014), these dipeptides could be interesting candidates for triggering metabolic shifts.

Under C restricted conditions imposed by longer night periods coupled with low light intensity, sugar and energy supply relies on autophagy to provide recycling metabolites and building blocks to sustain plant growth (Izumi et al., 2013). In addition, proteolysis is induced at night and considered one of the first responses when the C supply drops, provoking a change from protein synthesis to degradation to provide an alternative source of energy (Usadel et al., 2008; Ishihara et al., 2015). As 11 out of the 31 dipeptides oscillating solely under SD were previously either associated with autophagy or displayed lower levels in autophagy mutants (Thirumalaikumar et al., 2020), their oscillation under C limited supply might also result from this catabolic process. The massive increase of oscillating dipeptides in plants under SD photoperiod and the consequently reduced C mobilization compared with LD is thus suggested to be derived from induced protein degradation or autophagy, as these processes can take part in the biogenesis of small molecules (Thirumalaikumar et al., 2020), a hypothesis that remains to be tested. Besides the role of amino acids in protein synthesis, they are also required for signaling processes and can aid to balance the plant energy homeostasis under C deprivation. SD forces the metabolism to match reduced C mobilization with decreased C utilization, favoring C partitioning for respiration and maintenance to detriment of growth (Stitt and Zeeman, 2012). Along the time, several adaptations to SD such as C allocation, protein turnover and central metabolism allow the plant to continuously grow but at an extremely reduced rate due to C limitation (Smith and Stitt, 2007). Besides the differences of C supply related to the duration of the night, our experimental design does not exclude the effects of light intensity and photoperiod themselves in the dipeptides profile. The distinct light intensities were chosen to allow the comparison of plants at similar developmental stages and to reduce the gap in terms of daily light integral (DLI) between the two photoperiods. Accordingly, some dipeptides accumulated in high light conditions (LD, 300 compared with 150 μμE.m^–2^.sec^–1^) (Thirumalaikumar et al., 2020), but from the 31 dipeptides oscillating only in SD (the photoperiod condition with the highest light intensity in the present study), only five of them match this data (Thr-Glu, Val-Asp, Ala-Trp, Gly-Pro). Moreover, the combined increased Glu and Glu-containing dipeptides at the same time points in the dark might indicate altered N metabolism (Hildebrandt et al., 2015) along the day under limited C supplied for growth (SD), although dipeptides regulatory roles have shown to be diverse than the amino acid itself (Moreno et al., 2021).

The more relaxed energy management in LD photoperiod (Baerenfaller et al., 2015) might be associated with the decreased number of oscillating peptides compared to SD. A considerable number of the dipeptides oscillating under LD presented BCAA either at N- or at the C-terminus, mostly found in Cluster 2 (Figure 2). Interestingly, genes from the BCAA degradation pathways are upregulated during the night, when this catabolic activity is required to fulfill energy demand (Caldana et al., 2011; Peng et al., 2015) and what could have been in line with the night peak characteristic from this Cluster.

Plants facing contrasting C availability presented specific but integrated sugar signaling pathways. One example is the TOR/SnRK1 hub; high C availability relies on TOR, whilst low C supply requires SnRK1 to mediate metabolic responses (Wingler, 2018). TOR connects nutrients, inner inputs, and environmental signals to control several aspects of C metabolism (Dobrenel et al., 2013). In line with that, the most marked phenotype of *raptor1b* plants under high C availability strengthens the role of TOR as a fundamental adaptor to C supply. Despite the increasing evidence linking the TORC pathway to the integration of metabolic signals, energy status, and hormones in a wide range of plant growth-mediated processes (Moreau et al., 2012; Caldana et al., 2013; Xiong et al., 2013; Pfeiffer et al., 2016; Zhang et al., 2016; Dong et al., 2017; Li et al., 2017; Mohammed et al., 2018; Salem et al., 2018), little is known about its mode of action and the key players in controlling cellular homeostasis in a condition-dependent manner. The downregulation of TORC in plants activates autophagy, which derives from the generation of autophagosomes, enhanced expression of *ATG* genes, and their phosphorylation [reviewed by Mugume et al. (2020)], aside from nucleotides depletion (Kazibwe et al., 2020). The role of TOR as a repressor of autophagy (Pu et al., 2017), this process suggested to generate proteogenic dipeptides (Thirumalaikumar et al., 2020), makes *raptor1b* a suitable model to study the regulatory interplay among light, C availability, TOR, and small molecules. Compared with Col-0 *A. thaliana*, the increased number of altered dipeptides from the *raptor1b* plants grown under LD (Figure 3) might be linked to the de-activated autophagy in this mutant. Autophagy is repressed by TOR and activated by SnRK1, both proteins regulated by the nutrient availability in plants (Pu et al., 2017). Under nutrient deprivation, such as C shortage imposed by extended darkness period, TOR is inhibited *via* RAPTOR by SnRK1, thus releasing the repression of autophagy which will the outcome in recycling building blocks to relieve plant metabolism (Nukarinen et al., 2016; Soto-Burgos and Bassham, 2017). Not surprisingly, there was a larger number of dipeptides significantly increasing in *raptor1b* under LD conditions compared with Col-0. Since all the 16 dipeptides found to respond either to the reduced proportion of C available to growth imposed by SD conditions or the suppression of RAPTOR1B were glucogenic, it is reasonable to speculate that they could be involved in generating glucose to bypass possible C limitations. Out of these selected dipeptides, six and five presented Pro or BCAA at the N- or C-terminal. The generation of some of these dipeptides could be a product of autophagy, given that from the 16 selected dipeptides, 6 (Asp-Phe, Glu-Ala, Pro-Ala, Pro-Ser, Val-Gln, and Val-Asp) were either associated with increased autophagy transcripts or displayed reduced levels in autophagy mutants (Thirumalaikumar et al., 2020).

Regardless of the condition, Pro levels itself increased in *raptor1b* compared with Col-0 plants, which is a characteristic output of TORC inhibition (Moreau et al., 2012; Caldana et al., 2013; Salem et al., 2017; da Silva et al., 2021). This increment is even more pronounced when plants are transferred to a condition of augmented C supply (Moreau et al., 2012), matching the higher magnitude of phenotypic changes in *raptor1b* plants under LD (Salem et al., 2018). Pro accumulation is well known to occur under various stress conditions, probably playing an osmoprotective role (Verbruggen and Hermans, 2008). More recently, a new role for Pro in stimulating leaf nighttime O_2_ consumption rate under the control of the TOR pathway has emerged in a TOR-dependent context (O’Leary et al., 2020). The higher levels of some amino acids may require TORC to restrict respiratory catabolism preventing nutrient depletion. To overcome C deprivation conditions, such as long nights, the use of amino acids and fatty acids as respiratory substrates in plants can be a strategy to grant survival. Therefore, we favor the interpretation that the altered levels of Pro or Pro-containing dipeptides in *raptor1b* compared to Col-0 Arabidopsis might indicate that the mutants induce respiratory catabolism to feed C into the respiratory system; a hypothesis that demands further investigation to be proved. In addition to their role as putative respiratory substrates, we speculate that dipeptides would bind and regulate the activity of proteins. Nevertheless, as the TOR pathway integrates light and nutrient signals [reviewed by Wu et al. (2018)], besides the differences in C supply regarding SD and LD conditions, we cannot rule out the effects of both light intensity and duration on the alteration of these dipeptides’ levels in *raptor1b*.

Herein, we mined the SLIMP dataset for targets of a representative Pro-containing dipeptide, which we identified to be possibly related to C restriction. Dipeptides can influence the function of proteins and determined processes. Uptake of the dipeptide Gly-Sar can mediate mTOR activity in leukemia stem cells (Naka et al., 2015). Interestingly, several bacterial Pro-containing cyclodipeptides were proven to activate the TOR signaling pathway increasing growth in plants (Corona-Sánchez et al., 2019; González-López et al., 2021), including cyclodipeptides versions of Pro-Phe, which oscillated only in *raptor1b* (Supplementary Figures 2, 3) and Pro-Val, whose level was increased in the mutant compared to Col-0 under LD (Figure 3; Supplementary Figure 4). Among the 124 proteins identified as putative Pro-Gln interactors, we retrieved four related to the TOR pathway, besides several enzymes involved in C metabolism, such as glycolysis, gluconeogenesis, TCA cycle, and PPP (Figure 4). Threonine synthase (TS) and Tubulin alpha-6 (TUA6) are proteins coeluting with Pro-Gln that have been previously shown to physically interact with RAPTOR1B in protein-protein interaction assays (Van Leene et al., 2019). As we were interested in searching for proteins related to C metabolism, GAPC, G6PDH, 6PGDH, CSY2, CSY3, ICDHP, IMS1, and ILV5 were highlighted from this list. The G6PDH and 6PGDH are enzymes of the PPP downstream of glucose-6-phosphate that have an interplay with the TOR pathway in other organisms (Wagle et al., 1998; Tsouko et al., 2014). While CSY2 and CSY3 are required for fatty acid respiration (Pracharoenwattana et al., 2005), ICDHP catalyzes the conversion between isocitrate and 2-oxoglutarate (2OG). Analogously to G6PDH and 6PGDH, ICDHP is an NADP-dehydrogenase and contributes to cellular NADPH production (Leterrier et al., 2012). Interestingly, the IMS1 mutant *rol17* showed reduced sensitivity to the TOR inhibitor AZD8055, establishing a yet undepicted connection between this protein and the TOR network to adjust metabolic homeostasis (Schaufelberger et al., 2019). During C starvation, BCAAs support electron provision to electron-transfer flavoprotein/electron-transfer flavoprotein: ubiquinone oxidoreductase (ETF/ETFQO) complex, sustaining mitochondrial respiration under C starvation conditions (Araújo et al., 2010). Taken together, Pro-Gln interactors pointed out a role in providing alternative substrates for sustaining respiration under C limited conditions.

As no differential activity could be retrieved when Pro-Gln was added in the plant lysates (Supplementary Figure 5), we could not validate the role of this dipeptide in modulating the activities of GAPC, G6PDH, 6PGDH, and ICDHP. However, we cannot exclude that these results are dependent on the experimental conditions, such as the choice of the starting material, which might demand other players or might be inhibited in the specific conditions of the assay. Moreover, the obtained results do not exclude the binding, as not all interactional events will necessarily result in altered enzymatic activity. Future work will concentrate on the functional validation of these putative targets, which may be obtained by recombinant production and purification of these proteins to assay the enzymatic activity with no interferents.

## Data Availability Statement

The original contributions presented in the study are included in the article/Supplementary Material, further inquiries can be directed to the corresponding authors.

## Author Contributions

ML analyzed the mass spectrometry data and generated the Figures. MJC-R, ML, CC, and AS interpreted the data, wrote the manuscript, and revised it. CCM-B and RIM designed and performed the experiments, BZ generated SLIMP dataset using PROMIS data provided by the group of AS under supervision of ZN. AS and CC designed and supervised the experiments and the manuscript revision.

## Conflict of Interest

The authors declare that the research was conducted in the absence of any commercial or financial relationships that could be construed as a potential conflict of interest.

## Publisher’s Note

All claims expressed in this article are solely those of the authors and do not necessarily represent those of their affiliated organizations, or those of the publisher, the editors and the reviewers. Any product that may be evaluated in this article, or claim that may be made by its manufacturer, is not guaranteed or endorsed by the publisher.

## Footnotes

1
https://zenodo.org/badge/latestdoi/393474100

## Supplementary Material

Supplementary Figure 1 Oscillation of amino acid and dipeptide levels in *A. thaliana* Col-0 plants throughout a diel cycle (24 h). Dipeptides and amino acid levels that were significantly (adjusted *P*-value ≤ 0.05) affected throughout a diel cycle at **(A)** SD and **(B)** LD conditions are illustrated as a heatmap. Shown is scaled abundance. Euclidean distance was computed between the rows and row-wise clustering was performed using the complete method. *n* biological replicates = 3–5.

Supplementary Figure 2 Oscillation of amino acid and dipeptide levels in *A. thaliana raptor1b* plants throughout a diel cycle (24 h). Dipeptides and amino acid levels that were significantly (adjusted *P* value ≤ 0.05) affected throughout a diel cycle at **(A)** SD and LD, **(B)** solely at SD, and **(C)** solely at LD conditions are illustrated as a heatmap. Metabolites were ordered alphabetically. Shown is scaled abundance. Kmeans clustering of the metabolites levels at **(D)** SD and **(E)** LD was performed with k = 3. *n* biological replicates = 3–5.

Supplementary Figure 3 Oscillation of amino acid and dipeptide levels in *A. thaliana raptor1b* plants throughout a diel cycle (24 h). Dipeptides and amino acid levels that were significantly (adjusted *P*-value ≤ 0.05) affected throughout a diel cycle at **(A)** SD and **(B)** LD conditions are illustrated as a heatmap. Shown is scaled abundance. Euclidean distance was computed between the rows and row-wise clustering was performed using the complete method. *n* biological replicates = 3–5.

Supplementary Figure 4 Fold change values of the oscillating amino acid and dipeptide levels in *A. thaliana raptor1b* over Col-0 plants throughout a diel cycle (24 h). Fold change [log_2_] of the dipeptides and amino acid levels significantly (adjusted *P*-value ≤ 0.05) affected throughout a diel cycle at **(A)** SD and **(B)** LD conditions are illustrated as a heatmap. Euclidean distance was computed between the rows and row-wise clustering was performed using the complete method.

Supplementary Figure 5 Enzymatic activity measurements of Pro-Gln putative protein interactors. **(A)** ICDHP, **(B)** GAPC, **(C)** G6PDH, and **(D)** 6PGDH activity measurements under control conditions (mock) or with the addition of 100 μM Pro, Gln, or Pro-Gln. Enzymatic assays were performed on plant lysates harvested 2 h into the day (white bars) and 2 h into the dark (gray bars). Data are the mean of at least two independent data sets ± standard error.

Supplementary Table 1 One-way analysis of variance (ANOVA) followed by Bonferroni’s correction for multiple comparisons (.adj) of the intensities from the identified dipeptides and amino acids in Col-0 *A. thaliana* along a diel cycle under SD and LD conditions. The main effect of time (0–24 h) was analyzed under SD and LD conditions. Only the dipeptides showing significant *P*-values (*P* < 0.05) are presented. *n* biological replicates = 3–5.

Supplementary Table 2 One-way ANOVA followed by Bonferroni’s correction for multiple comparisons (.adj) of the intensities from the identified dipeptides and amino acids in *raptor1b A. thaliana* along a diel cycle under SD and LD conditions. The main effect of time (0–24 h) was analyzed under SD and LD conditions. Only the dipeptides showing significant *P*-values (*P* < 0.05) are presented. *n* biological replicates = 3–5.

Supplementary Table 3 Two-way ANOVA followed by Bonferroni’s correction for multiple comparisons (.adj) of the intensities from the identified dipeptides and amino acids. The main effects of time (0–24 h), *raptor1b* genotype (over Col-0), or both factors were analyzed under SD and LD conditions. Significant *P*-values are highlighted in red (*P* < 0.05). *n* biological replicates = 3–5.

Supplementary Table 4 Statistical summary of the measured amino acids and dipeptides in Col-0 and *raptor1b A. thaliana* under SD. *n* biological replicates = 3–5.

Supplementary Table 5 Statistical summary of the measured amino acids and dipeptides in Col-0 and *raptor1b A. thaliana* under LD. *n* biological replicates = 3–5.

Supplementary Table 6 Pro-Gln protein interactors retrieved from SLIMP (Zühlke et al., 2021), used for STRING (Szklarczyk et al., 2016) protein-protein interaction analysis. Targets mentioned in the text are highlighted in red letters.

Supplementary Table 7 Parameters used in Cytoscape (Shannon et al., 2003) to build the interaction network of the Pro-Gln interactors retrieved from SLIMP (Zühlke et al., 2021) in Cytoscape. Protein-protein interactions were imported from STRING (Szklarczyk et al., 2016) based on the experimental and database evidence.

Supplementary Dataset 1 Liquid chromatography-mass spectrometry (LC-MS) measurements of amino acids and dipeptides from the methyl-tert-butyl-ether (MTBE) -extraction in Col-0 and *raptor1b A. thaliana* along a diel cycle under SD condition. Values are normalized as described in the “Materials and Methods” section. *n* biological replicates = 3–5.

Supplementary Dataset 2 LC-MS measurements of the polar fraction from the MTBE-extraction in Col-0 and *raptor1b A. thaliana* along a diel cycle under SD condition. Raw chromatograms were processed using Expressionist Refiner MS as described in the “Materials and Methods” section. *n* biological replicates = 3–5.

Supplementary Dataset 3 LC-MS measurements of amino acids and dipeptides from the MTBE-extraction in Col-0 and *raptor1b A. thaliana* along a diel cycle under LD condition. Values are normalized as described in the “Materials and Methods” section. *n* biological replicates = 3–5.

Supplementary Dataset 4 LC-MS measurements of polar fraction from the MTBE-extraction in Col-0 and *raptor1b A. thaliana* along a diel cycle under LD condition. Raw chromatograms were processed using Expressionist Refiner MS as described in the “Materials and Methods” section. *n* biological replicates = 3–5.

## References

[B1] Agredano-Moreno L. T. Reyes de la Cruz H. Martínez-Castilla L. P. Sánchez de Jiménez E. (2007). Distinctive expression and functional regulation of the maize (*Zea mays* L.) TOR kinase ortholog. *Mol. BioSyst.* 3 794–794. 10.1039/b705803a 17940662

[B2] Alves L. C. Llerena J. P. P. Mazzafera P. Vicentini R. (2019). Diel oscillations in cell wall components and soluble sugars as a response to short-day in sugarcane (*Saccharum* sp.). *BMC Plant Biol.* 19:215. 10.1186/s12870-019-1837-4 31122198 PMC6533765

[B3] Araújo W. L. Ishizaki K. Nunes-Nesi A. Larson T. R. Tohge T. Krahnert I. (2010). Identification of the 2-hydroxyglutarate and isovaleryl-CoA dehydrogenases as alternative electron donors linking lysine catabolism to the electron transport chain of Arabidopsis mitochondria. *Plant Cell* 22, 1549–1563. 10.1105/tpc.110.075630 20501910 PMC2899879

[B4] Baerenfaller K. Massonnet C. Hennig L. Russenberger D. Sulpice R. Walsh S. (2015). A long photoperiod relaxes energy management in *Arabidopsis* leaf six. *Curr. Plant Biol.* 2 34–45. 10.1016/J.CPB.2015.07.001

[B5] Busche M. Scarpin M. R. Hnasko R. Brunkard J. O. (2021). TOR coordinates nucleotide availability with ribosome biogenesis in plants. *Plant Cell* 33 1615–1632. 10.1093/plcell/koab043 33793860 PMC8254494

[B6] Caldana C. Degenkolbe T. Cuadros-Inostroza A. Klie S. Sulpice R. Leisse A. (2011). High-density kinetic analysis of the metabolomic and transcriptomic response of *Arabidopsis* to eight environmental conditions. *Plant J.* 67 869–884. 10.1111/j.1365-313X.2011.04640.x 21575090

[B7] Caldana C. Li Y. Leisse A. Zhang Y. Bartholomaeus L. Fernie A. R. (2013). Systemic analysis of inducible target of rapamycin mutants reveal a general metabolic switch controlling growth in *Arabidopsis thaliana*. *Plant J.* 73 897–909. 10.1111/tpj.12080 23173928

[B8] Caldana C. Martins M. C. M. Mubeen U. Urrea-Castellanos R. (2019). The magic “hammer” of TOR: the multiple faces of a single pathway in the metabolic regulation of plant growth and development. *J. Exp. Bot.* 70 2217–2225. 10.1093/jxb/ery459 30722050

[B9] Corona-Sánchez I. Peña-Uribe C. A. González-López O. Villegas J. Campos-Garcia J. de la Cruz H. R. (2019). Cyclodipeptides from *Pseudomonas aeruginosa* modulate the maize (*Zea mays* L.) root system and promote S6 ribosomal protein kinase activation. *PeerJ* 7:e7494. 10.7717/peerj.7494 31523498 PMC6717507

[B10] da Silva V. C. H. Martins M. C. M. Calderan-Rodrigues M. J. Artins A. Monte Bello C. C. Gupta S. (2021). Shedding light on the dynamic role of the “Target of Rapamycin” kinase in the fast-growing C4 species *Setaria viridis*, a suitable model for biomass crops. *Front. Plant Sci.* 12:637508. 10.3389/fpls.2021.637508 33927734 PMC8078139

[B11] Dardente H. Hazlerigg D. G. Ebling F. J. (2014). Thyroid hormone and seasonal rhythmicity. *Front. Endocrinol*. 26:19. 10.3389/fendo.2014.00019 24616714 PMC3935485

[B12] Deprost D. Yao L. Sormani R. Moreau M. Leterreux G. Nicolai M. (2007). The Arabidopsis TOR kinase links plant growth, yield, stress resistance and mRNA translation. *EMBO Rep.* 8 864–870. 10.1038/sj.embor.7401043 17721444 PMC1973950

[B13] Dobrenel T. Caldana C. Hanson J. Robaglia C. Vincentz M. Veit B. (2016). TOR signaling and nutrient sensing. *Ann. Rev. Plant Biol.* 67 261–285. 10.1146/annurev-arplant-043014-114648 26905651

[B14] Dobrenel T. Marchive C. Azzopardi M. Clément G. Moreau M. Sormani R. (2013). Sugar metabolism and the plant target of rapamycin kinase: a sweet operaTOR? *Front. Plant Sci.* 4:93. 10.3389/fpls.2013.00093 23641244 PMC3640205

[B15] Dong Y. Silbermann M. Speiser A. Forieri I. Linster E. Poschet G. (2017). Sulfur availability regulates plant growth *via* glucose-TOR signaling. *Nat. Commun.* 8:1174. 10.1038/s41467-017-01224-w 29079776 PMC5660089

[B16] Doppler M. Kluger B. Bueschl C. Steiner B. Buerstmayr H. Lemmens M. (2019). Stable isotope-assisted plant metabolomics: investigation of phenylalanine-related metabolic response in wheat upon treatment with the Fusarium Virulence Factor deoxynivalenol. *Front Plant Sci.* 10:1137. 10.3389/fpls.2019.01137 31736983 PMC6831647

[B17] Doust A. N. (2017). “The effect of photoperiod on flowering time, plant architecture, and biomass in Setaria in the genetics and genomics of Setaria,” in *Plant Genetics and Genomics: Crops and Models*, eds Doust A. N. Diao X. M. (New York, NY: Springer-Verlag), 19.

[B18] Eastmond P. J. Astley H. M. Parsley K. Aubry S. Williams B. P. Menard G. N. (2015). *Arabidopsis* uses two gluconeogenic gateways for organic acids to fuel seedling establishment. *Nat. Commun.* 6:6659. 10.1038/ncomms7659 25858700 PMC4403315

[B19] Garner W. W. Allard H. A. (1920). Effect of the relative length of day and night and other factors of the environment on growth and reproduction in plants. *J. Agric Res.* 18 553–606. 10.1175/1520-0493192048<415b:EOTRLO<2.0.CO;2

[B20] Giavalisco P. Li Y. Matthes A. Eckhardt A. Hubberten H.-M. Hesse H. (2011). Elemental formula annotation of polar and lipophilic metabolites using 13 C, 15 N and 34 S isotope labelling, in combination with high-resolution mass spectrometry. *Plant J.* 68 364–376. 10.1111/j.1365-313X.2011.04682.x 21699588

[B21] Gibon Y. Blaesing O. E. Hannemann J. Carillo P. Höhne M. Hendriks J. H. M. (2004). A robot-based platform to measure multiple enzyme activities in *Arabidopsis* using a set of cycling assays: comparison of changes of enzyme activities and transcript levels during diurnal cycles and in prolonged darkness. *Plant Cell* 16 3304–3325. 10.1105/tpc.104.025973 15548738 PMC535875

[B22] González-López O. Palacios-Nava B. B. Peña-Uribe C. A. Campos-García J. López-Bucio J. García-Pineda E. (2021). Growth promotion in *Arabidopsis thaliana* by bacterial cyclodipeptides involves the TOR/S6K pathway activation. *J. Plant Physiol.* 257:153343. 10.1016/j.jplph.2020.153343 33387853

[B23] Hara K. Maruki Y. Long X. Yoshino K.-i. Oshiro N. Hidayat S. (2002). Raptor, a binding partner of target of rapamycin (TOR), mediates TOR action. *Cell* 110 177–189. 10.1016/s0092-8674(02)00833-4 12150926

[B24] Hildebrandt T. M. Nunes Nesi A. Araújo W. L. Braun H. P. (2015). Amino acid catabolism in plants. *Mol. Plant*. 8 1563–1579. 10.1016/j.molp.2015.09.005 26384576

[B25] Ishihara H. Moraes T. A. Pyl E.-T. Schulze W. S. Obata T. Scheffel A. (2017). Growth rate correlates negatively with protein turnover in *Arabidopsis* accessions. *Plant J.* 91 416–429. 10.1111/tpj.13576 28419597

[B26] Ishihara H. Obata T. Sulpice R. Fernie A. R. Stitt M. (2015). Quantifying protein synthesis and degradation in *Arabidopsis* by dynamic ^13^CO_2_ labeling and analysis of enrichment in individual amino acids in their free pools and in protein. *Plant Phys.* 168 74–93. 10.1104/pp.15.00209 25810096 PMC4424029

[B27] Izumi M. Hidema J. Makino A. Ishida H. (2013). Autophagy contributes to nighttime energy availability for growth in *Arabidopsis*. *Plant Physiol.* 161 1682–1693. 10.1104/pp.113.215632 23457226 PMC3613448

[B28] Jeske L. Placzek S. Schomburg I. Chang A. Schomburg D. (2019). BRENDA in 2019: a European ELIXIR core data resource. *Nucleic Acids Res.* 47 D542–D549. 10.1093/nar/gky1048 30395242 PMC6323942

[B29] Jüppner J. Mubeen U. Leisse A. Caldana C. Wiszniewski A. Steinhauser D. (2018). The target of rapamycin kinase affects biomass accumulation and cell cycle progression by altering carbon/nitrogen balance in synchronized *Chlamydomonas reinhardtii* cells. *Plant J.* 93, 355–376. 10.1111/tpj.13787 29172247

[B30] Kazibwe Z. Soto-Burgos J. MacIntosh G. C. Bassham D. C. (2020). TOR mediates the autophagy response to altered nucleotide homeostasis in an RNase mutant. *J. Exp. Bot*. 71 6907–6920. 10.1093/jxb/eraa410 32905584

[B31] Khoeyi Z. A. Seyfabadi J. Ramezanpur Z. (2012). Effect of light intensity and photoperiod on biomass and fatty acids. *Aquacult. Int.* 20 41–49. 10.1007/s10499-011-9440-1

[B32] Kim D.-H. Sarbassov D. D. Ali S. M. King J. E. Latek R. R. Erdjument-Bromage H. (2002). mTOR interacts with raptor to form a nutrient-sensitive complex that signals to the cell growth machinery. *Cell* 110 163–175. 10.1016/s0092-8674(02)00808-5 12150925

[B33] Kuhn M. von Mering C. Campillos M. Jensen L. J. Bork P. (2008). STITCH: interaction networks of chemicals and proteins. *Nucleic Acids Res.* 36 D684–D688. 10.1093/nar/gkm795 18084021 PMC2238848

[B34] Leterrier M. Juan B. Barroso J. B. Valderrama R. Palma J. M. Corpas F. J. (2012). NADP-dependent isocitrate dehydrogenase from *Arabidopsis* roots contributes in the mechanism of defence against the nitro-oxidative stress induced by salinity. *Sci. World J.* 2:694740. 10.1100/2012/694740 22649311 PMC3354597

[B35] Li X. Cai W. Liu Y. Li H. Fu L. Liu Z. (2017). Differential TOR activation and cell proliferation in *Arabidopsis* root and shoot apexes. *Proc. Natl. Acad. Sci. U.S.A.* 114 2765–2770. 10.1073/pnas.1618782114 28223530 PMC5347562

[B36] Liu D. L. Y. Christians N. E. (1994). Isolation and identification of root-inhibiting compounds from corn gluten hydrolysate. *J. Plant Growth Regul.* 13 227–230. 10.1007/BF00226041

[B37] Liu G. Y. Sabatini D. M. (2020). mTOR at the nexus of nutrition, growth, ageing and disease. *Nat. Rev. Mol. Cell Biol.* 21 183–203. 10.1038/s41580-019-0199-y 31937935 PMC7102936

[B38] Liu Y. Bassham D. C. (2010). TOR is a negative regulator of autophagy in *Arabidopsis thaliana*. *PLoS One* 5:e11883. 10.1371/journal.pone.0011883 20686696 PMC2912371

[B39] Liu Y. Duan X. Zhao X. Ding W. Wang Y. Xiong Y. (2021). Diverse nitrogen signals activate convergent ROP2-TOR signaling in *Arabidopsis*. *Dev Cell* 56 1283–1295. 10.1016/j.devcel.2021.03.022 33831352

[B40] Luzarowski M. Vicente R. Kiselev A. Wagner M. Schlossarek D. Erban A. (2021). Global mapping of protein–metabolite interactions in *Saccharomyces cerevisiae* reveals that Ser-Leu dipeptide regulates phosphoglycerate kinase activity. *Commun. Biol.* 4:181. 10.1038/s42003-021-01684-3 33568709 PMC7876005

[B41] Maegawa K. Takii R. Ushimaru T. Kozaki A. (2015). Evolutionary conservation of TORC1 components, TOR, Raptor, and LST8, between rice and yeast. *Mol. Genet. Genom.* 290 2019–2030. 10.1007/s00438-015-1056-0 25956502

[B42] Mahfouz M. M. Kim S. Delauney A. J. Verma D. P. S. (2006). Arabidopsis TARGET OF RAPAMYCIN interacts with RAPTOR, which regulates the activity of S6 Kinase in response to osmotic stress signals. *Plant Cell* 18 477–490. 10.1105/tpc.105.035931 16377759 PMC1356553

[B43] Menand B. Desnos T. Nussaume L. Berger F. Bouchez D. Meyer C. (2002). Expression and disruption of the *Arabidopsis* TOR (Target of Rapamycin) gene. *Proc. Natl. Acad. Sci. U.S.A.* 99 6422–6427. 10.1073/pnas.092141899 11983923 PMC122964

[B44] Mengin V. Pyl E.-T. Moraes T. A. Sulpice R. Krohn N. Encke B. (2017). Photosynthate partitioning to starch in *Arabidopsis thaliana* is insensitive to light intensity but sensitive to photoperiod due to a restriction on growth in the light in short photoperiods. *Plant Cell Environ.* 40 2608–2627. 10.1111/pce.13000 28628949

[B45] Mohammed B. Bilooei S. F. Doczi R. Grove E. Railo S. Palme K. (2018). Converging light, energy and hormonal signaling control meristem activity, leaf initiation, and growth. *Plant Physiol.* 176 1365–1381. 10.1104/pp.17.01730 29284741 PMC5813583

[B46] Monte-Bello C. C. Araujo E. F. Martins M. C. M. Mafra V. da Silva V. C. H. Celente V. (2018). A flexible low cost hydroponic system for assessing plant responses to small molecules in sterile conditions. *JOVE* 138:57800. 10.3791/57800 30199012 PMC6231878

[B47] Moraes T. A. Mengin V. Annunziata M. G. Encke B. Krohn N. Höhne M. (2019). Response of the circadian clock and diel starch turnover to one day of low light or low CO_2_. *Plant Physiol.* 179 1457–1478. 10.1104/pp.18.01418 30670603 PMC6446786

[B48] Moreau M. Azzopardi M. Clément G. Dobrenel T. Marchive C. Renne C. (2012). Mutations in the Arabidopsis homolog of LST8/GßL, a partner of the Target of Rapamycin kinase, impair plant growth, flowering, and metabolic adaptation to long days. *Plant Cell* 24 463–481. 10.1105/tpc.111.091306 22307851 PMC3315227

[B49] Moreno J. C. Rojas B. E. Vicente R. Gorka M. Matz T. Chodasiewicz M. (2021). Tyr-Asp inhibition of glyceraldehyde 3-phosphate dehydrogenase affects plant redox metabolism. *EMBO J.* 40:e106800. 10.15252/embj.2020106800 34156108 PMC8327957

[B50] Mubeen U. Giavalisco P. Caldana C. (2019). TOR inhibition interrupts the metabolic homeostasis by shifting the carbon-nitrogen balance in *Chlamydomonas reinhardtii*. *Plant Signal Behav.* 14 1670595–1670595. 10.1080/15592324.2019.1670595 31583958 PMC6804693

[B51] Mugume Y. Kazibwe Z. Bassham D. (2020). Target of rapamycin in control of autophagy: puppet master and signal integrator. *IJMS* 21:8259. 10.3390/ijms21218259 33158137 PMC7672647

[B52] Naka K. Jomen Y. Ishihara K. Kim J. Ishimoto T. Bae E. J. (2015). Dipeptide species regulate p38MAPKSmad3 signalling to maintain chronic myelogenous leukaemia stem cells. *Nat. Commun.* 6:8039. 10.1038/ncomms9039 26289811 PMC4560789

[B53] Nozue K. Covington M. F. Duek P. D. Lorrain S. Fankhauser C. Harmer S. L. (2007). Rhythmic growth explained by coincidence between internal and external cues. *Nature* 448 358–361. 10.1038/nature05946 17589502

[B54] Nukarinen E. Nägele T. Pedrotti L. Wurzinger B. Mair A. Landgraf R. (2016). Quantitative phosphoproteomics reveals the role of the AMPK plant ortholog SnRK1 as a metabolic master regulator under energy deprivation. *Sci. Rep.* 6:31697. 10.1038/srep31697 27545962 PMC4992866

[B55] O’Leary B. M. Oh G. G. K. Lee C. P. Millar A. H. (2020). Metabolite regulatory interactions control plant respiratory metabolism via target of rapamycin (TOR) kinase activation. *Plant Cell* 32, 666–682. 10.1105/tpc.19.00157 31888967 PMC7054028

[B56] Peng C. Uygun S. Shiu S. H. Last R. L. (2015). The impact of the branched-chain ketoacid dehydrogenase complex on amino acid homeostasis in *Arabidopsis*. *Plant Physiol.* 169 1807–1820. 10.1104/pp.15.00461 25986129 PMC4634046

[B57] Pfeiffer A. Janocha D. Dong Y. Medzihradszky A. Schöne S. Daum G. (2016). Integration of light and metabolic signals for stem cell activation at the shoot apical meristem. *eLife* 5:17023. 10.7554/eLife.17023 27400267 PMC4969040

[B58] Piao S. Wang X. Park T. Chen C. Lian X. He Y. (2019). Characteristics, drivers and feedbacks of global greening. *Nat. Rev. Earth Environ.* 1 14–27. 10.1038/s43017-019-0001-x.pdf

[B59] Pracharoenwattana I. Cornah J. E. Smith S. M. (2005). Arabidopsis peroxisomal citrate synthase is required for fatty acid respiration and seed germination. *Plant Cell* 17 2037–2048. 10.1105/tpc.105.031856 15923350 PMC1167550

[B60] Pu Y. Luo X. Bassham D. C. (2017). TOR-dependent and -independent pathways regulate autophagy in *Arabidopsis thaliana*. *Front. Plant Sci.* 8:1204. 10.3389/fpls.2017.01204 28744293 PMC5504165

[B61] Ren M. Qiu S. Venglat P. Xiang D. Feng L. Selvaraj G. (2011). Target of rapamycin regulates development and ribosomal RNA expression through kinase domain in *Arabidopsis*. *Plant Physiol.* 155 1367–1382. 10.1104/pp.110.169045 21266656 PMC3046592

[B62] Rius S. P. Casati P. Iglesias A. A. Gomez-Casati D. F. (2006). Characterization of an *Arabidopsis thaliana* mutant lacking a cytosolic non-phosphorylating glyceraldehyde-3-phosphate dehydrogenase. *Plant Mol. Biol*. 61 945–957. 10.1007/s11103-006-0060-5 16927206

[B63] Sadka A. Dahan E. Or E. Cohen L. (2000). NADP^+^-isocitrate dehydrogenase gene expression and isozyme activity during citrus fruit development. *Plant Sci*. 158 173–181. 10.1016/s0168-9452(00)00328-9 10996257

[B64] Salazar J. D. Saithong T. Brown P. E. Foreman J. Locke J. C. W. Halliday K. J. (2009). Prediction of photoperiodic regulators from quantitative gene circuit models. *Cell* 139 1170–1179. 10.1016/j.cell.2009.11.029 20005809

[B65] Salem M. A. Li Y. Bajdzienko K. Fisahn J. Watanabe M. Hoefgen R. (2018). RAPTOR controls developmental growth transitions by altering the hormonal and metabolic balance. *Plant Physiol.* 177 565–593. 10.1104/pp.17.01711 29686055 PMC6001337

[B66] Salem M. A. Li Y. Wiszniewski A. Giavalisco P. (2017). Regulatory-associated protein of TOR (RAPTOR) alters the hormonal and metabolic composition of *Arabidopsis* seeds, controlling seed morphology, viability and germination potential. *Plant J* 92 525–545. 10.1111/tpj.13667 28845535

[B67] Schaufelberger M. Galbier F. Herger A. Francisco R. B. Roffler S. Clement G. (2019). Mutations in the *Arabidopsis ROL17/isopropylmalate synthase 1* locus alter amino acid content, modify the TOR network, and suppress the root hair cell development mutant *lrx1*. *J. Exp. Bot.* 70 2313–2323. 10.1093/jxb/ery463 30753668 PMC6463047

[B68] Seaton D. D. Graf A. Baerenfaller K. Stitt M. Millar A. J. Gruissem W. (2018). Photoperiodic control of the *Arabidopsis* proteome reveals a translational coincidence mechanism. *Mol. Syst. Biol.* 14:e7962. 10.15252/msb.20177962 29496885 PMC5830654

[B69] Shannon P. Markiel A. Ozier O. Baliga N. S. Wang J. T. Ramage D. (2003). Cytoscape: a software environment for integrated models of biomolecular interaction networks. *Genome Res.* 13 2498–2504. 10.1101/gr.1239303 14597658 PMC403769

[B70] Smith A. M. Stitt M. (2007). Coordination of carbon supply and plant growth. *Plant Cell Environ.* 30 1126–1149. 10.1111/j.1365-3040.2007.01708.x 17661751

[B71] Sokolowska E. W. Schlossarek D. Luzarowski M. Skirycz A. (2019). PROMIS: global analysis of PROtein-metabolite interactions. *Curr. Protoc. Plant Biol.* 4:e20101. 10.1002/cppb.20101 31750999

[B72] Soto-Burgos J. Bassham D. C. (2017). SnRK1 activates autophagy *via* the TOR signaling pathway in *Arabidopsis thaliana*. *PLoS One* 12:e0182591. 10.1371/journal.pone.0182591 28783755 PMC5544219

[B73] Stitt M. Zeeman S. C. (2012). Starch turnover: pathways, regulation and role in growth. *Curr. Opin. Plant Biol.* 15 282–292. 10.1016/j.pbi.2012.03.016 22541711

[B74] Strehmel N. Hoehenwarter W. Mönchgesang S. Majovsky P. Krüger S. Scheel D. (2017). Stress-related mitogen-activated protein kinases stimulate the accumulation of small molecules and proteins in *Arabidopsis thaliana* root exudates. *Front. Plant Sci.* 21:1292. 10.3389/fpls.2017.01292 28785276 PMC5520323

[B75] Sulpice R. Flis A. Ivakov A. A. Apelt F. Krohn N. Encke B. (2014). Arabidopsis coordinates the diurnal regulation of carbon allocation and growth across a wide range of photoperiods. *Mol. Plant* 7 137–155. 10.1093/mp/sst127 24121291

[B76] Szklarczyk D. Morris J. H. Cook H. Kuhn M. Wyder S. Simonovic M. (2016). The STRING database in 2017: quality-controlled protein–protein association networks, made broadly accessible. *Nuclic Acids Res.* 45 D362–D368. 10.1093/nar/gkw937 27924014 PMC5210637

[B77] Thirumalaikumar V. P. Wagner M. Balazadeh S. Skirycz A. (2020). Autophagy is responsible for the accumulation of proteogenic dipeptides in response to heat stress in *Arabidopsis thaliana*. *FEBS J.* 288 281–292. 10.1111/febs.15336 32301545

[B78] Tsouko E. Khan A. S. White M. A. Han J. J. Shi Y. Merchant F. A. (2014). Regulation of the pentose phosphate pathway by an androgen receptor-mTOR-mediated mechanism and its role in prostate cancer cell growth. *Oncogenesis* 3:e103. 10.1038/oncsis.2014.18 24861463 PMC4035695

[B79] Usadel B. Bläsing O. E. Gibon Y. Retzlaff K. Höhne M. Günther M. (2008). Global Transcript levels respond to small changes of the carbon status during progressive exhaustion of carbohydrates in *Arabidopsis* rosettes. *Plant Phys* 146 1834–1861. 10.1104/pp.107.115592 18305208 PMC2287354

[B80] Van Leene J. Han C. Gadeyne A. Eeckhout D. Matthijs C. Cannoot B. (2019). Capturing the phosphorylation and protein interaction landscape of the plant TOR kinase. *Nat. Plants* 5 316–327. 10.1038/s41477-019-0378-z 30833711

[B81] Verbruggen N. Hermans C. (2008). Proline accumulation in plants: a review. *Amino Acids* 35 753–759. 10.1007/s00726-008-0061-6 18379856

[B82] Veyel D. Kierszniowska S. Kosmacz M. Sokolowska E. M. Michaelis A. Luzarowski M. (2017). System-wide detection of protein-small molecule complexes suggests extensive metabolite regulation in plants. *Sci. Rep.* 7:42387. 10.1038/srep42387 28205532 PMC5304321

[B83] Veyel D. Sokolowska E. M. Moreno J. C. Kierszniowska S. Cichon J. Wojciechowska I. (2018). PROMIS, global analysis of PROtein–metabolite interactions using size separation in *Arabidopsis thaliana*. *J. Biol. Chem.* 293 12440–12453. 10.1074/jbc.ra118.003351 29853640 PMC6093232

[B84] Wagle A. Jivraj S. Garlock G. L. Stapleton S. R. (1998). Insulin regulation of glucose-6-phosphate dehydrogenase gene expression is rapamycin-sensitive and requires phosphatidylinositol 3-Kinase. *J. Biol. Chem.* 273 14968–14974. 10.1074/jbc.273.24.14968 9614103

[B85] Wingler A. (2018). Transitioning to the next phase: the role of sugar signaling throughout the plant life cycle. *Plant Physiol.* 176 1075–1084. 10.1104/pp.17.01229 28974627 PMC5813577

[B86] Wu Y. Shi L. Fu L. Liu Y. Xiong Y. Sheen J. (2018). Integration of nutrient, energy, light, and hormone signaling *via* TOR in plants. *J. Exp. Bot.* 70 2227–2238. 10.1093/jxb/erz028 30715492 PMC6463029

[B87] Xiong Y. McCormack M. Li L. Hall Q. Xiang C. Sheen J. (2013). Glucose-TOR signalling reprograms the transcriptome and activates meristems. *Nature* 496 181–186. 10.1038/nature12030 23542588 PMC4140196

[B88] Yanovsky M. J. Kay S. A. (2002). Molecular basis of seasonal time measurement in *Arabidopsis*. *Nature* 419 308–312. 10.1038/nature00996 12239570

[B89] Zhang Z. Zhu J.-Y. Roh J. Marchive C. Kim S.-K. Meyer C. (2016). TOR signaling promotes accumulation of BZR1 to balance growth with carbon availability in *Arabidopsis*. *Curr. Biol.* 26 1854–1860. 10.1016/j.cub.2016.05.005 27345161 PMC5126233

[B90] Zühlke B. M. Sokolowska E. M. Luzarowski M. Schlossarek D. Chodasiewicz M. Leniak E. (2021). SLIMP: supervised learning of metabolite-protein interactions from co-fractionation mass spectrometry data. *bioRxiv* [Preprint]. 10.1101/2021.06.16.448636

