# Supplementary figures and images for: Proteogenic Dipeptides Are Characterized by Diel Fluctuations and Target of Rapamycin Complex-Signaling Dependency in the Model Plant Arabidopsis thaliana
Source: Front Plant Sci. 2021 Dec 22;12:758933. doi: 10.3389/fpls.2021.758933 (PMC8727597; doi:10.3389/fpls.2021.758933)

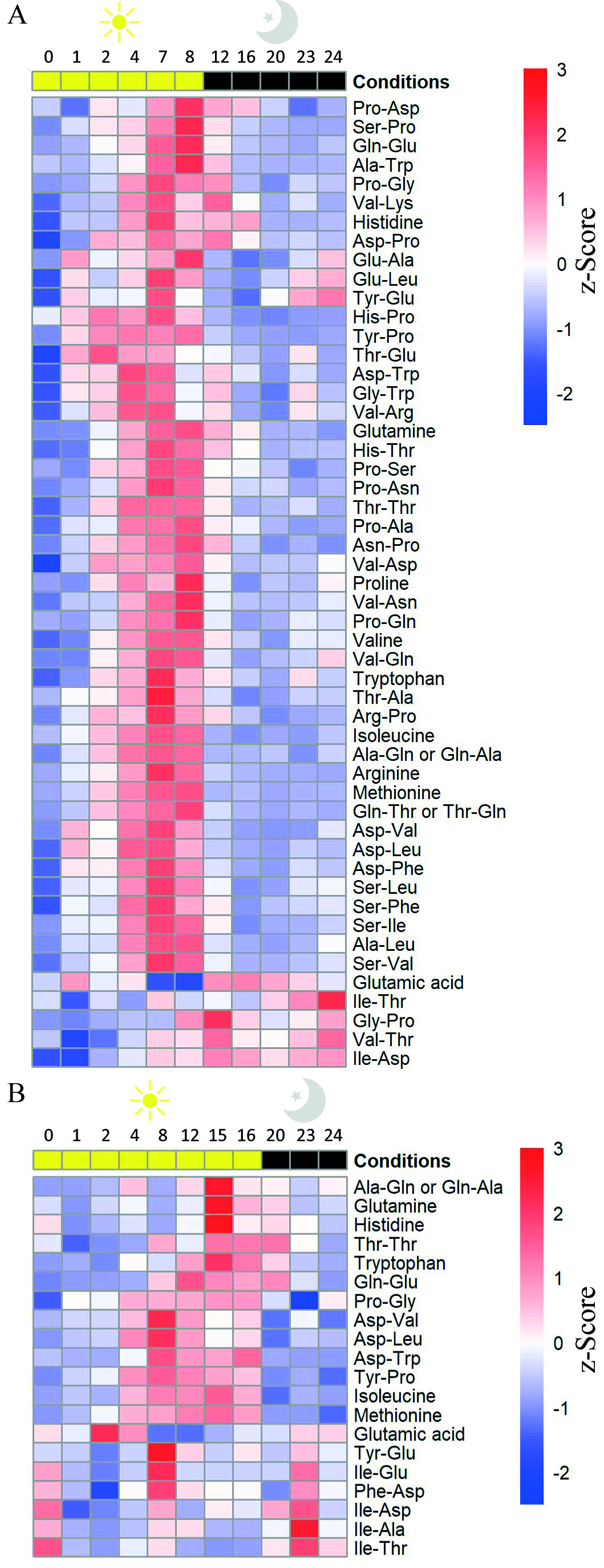

Supplement: Supplementary Figure 1 — Oscillation of amino acid and dipeptide levels in A. thaliana Col-0 plants throughout a diel cycle (24 h). Dipeptides and amino acid levels that were significantly (adjusted P-value ≤ 0.05) affected throughout a diel cycle at (A) SD and (B) LD conditions are illustrated as a heatmap. Shown is scaled abundance. Euclidean distance was computed between the rows and row-wise clustering was performed using the complete method. n biological replicates = 3–5. [file Image_1.TIF]

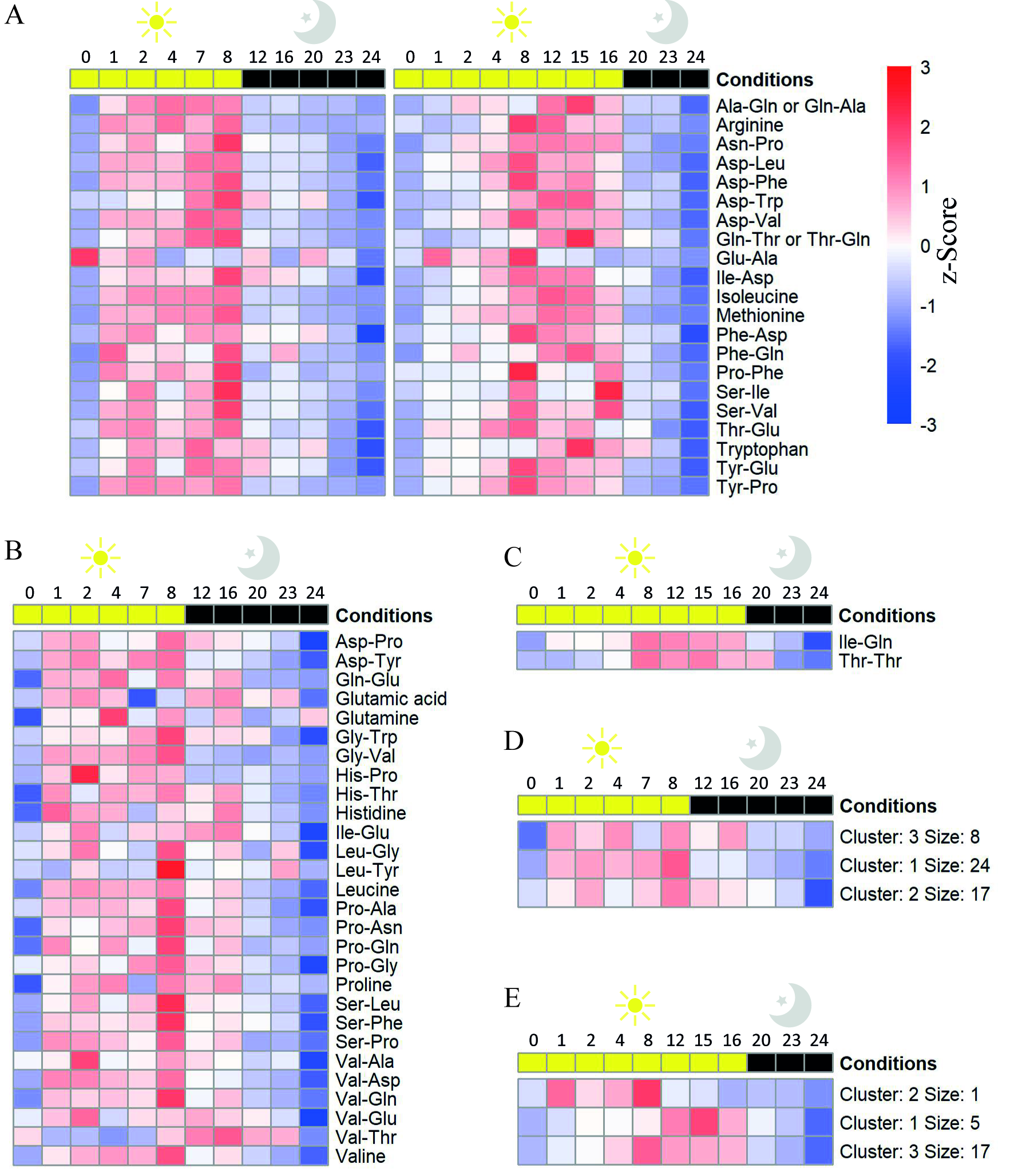

Supplement: Supplementary Figure 2 — Oscillation of amino acid and dipeptide levels in A. thaliana raptor1b plants throughout a diel cycle (24 h). Dipeptides and amino acid levels that were significantly (adjusted P value ≤ 0.05) affected throughout a diel cycle at (A) SD and LD, (B) solely at SD, and (C) solely at LD conditions are illustrated as a heatmap. Metabolites were ordered alphabetically. Shown is scaled abundance. Kmeans clustering of the metabolites levels at (D) SD and (E) LD was performed with k = 3. n biological replicates = 3–5. [file Image_2.TIF]

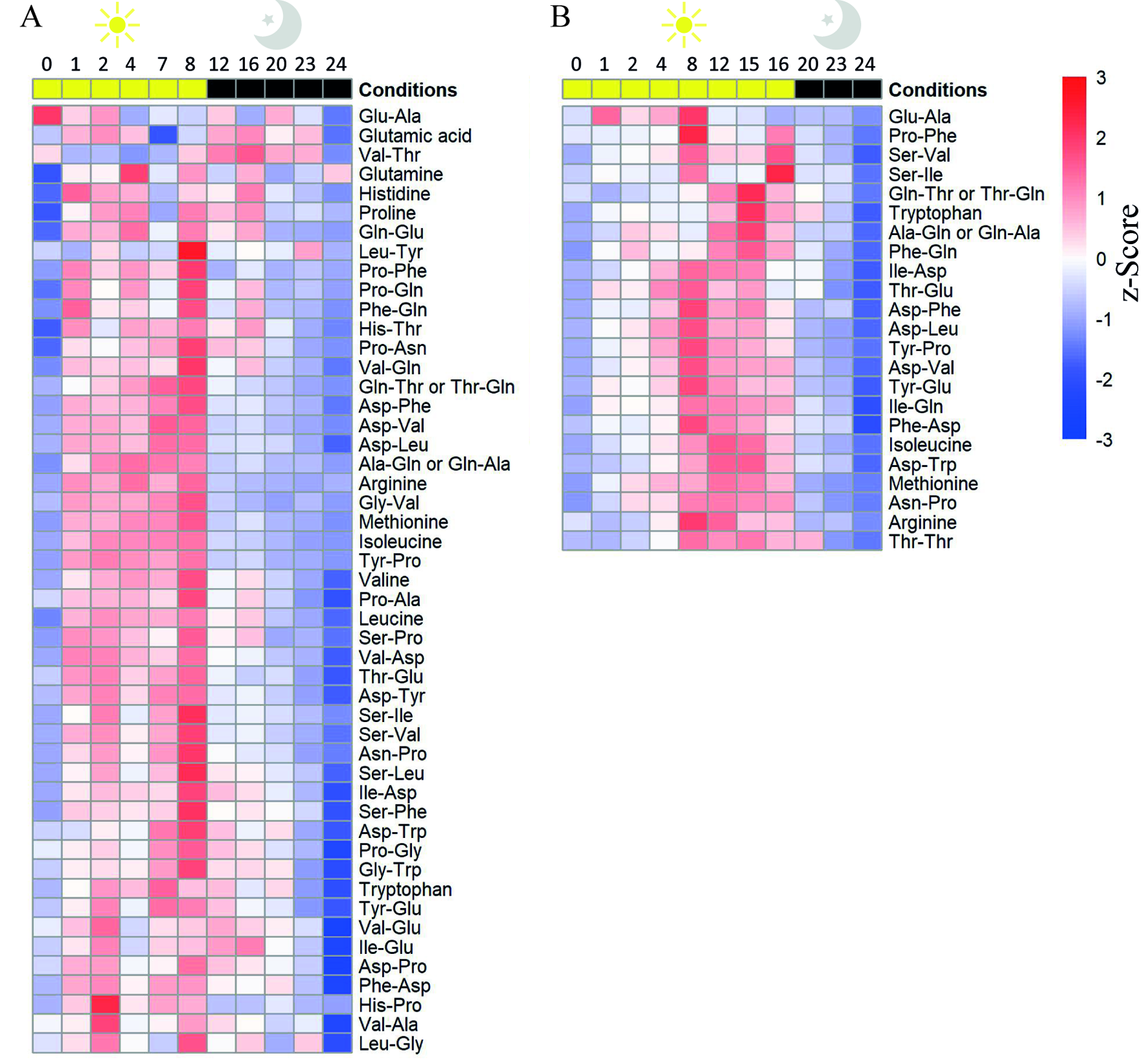

Supplement: Supplementary Figure 3 — Oscillation of amino acid and dipeptide levels in A. thaliana raptor1b plants throughout a diel cycle (24 h). Dipeptides and amino acid levels that were significantly (adjusted P-value ≤ 0.05) affected throughout a diel cycle at (A) SD and (B) LD conditions are illustrated as a heatmap. Shown is scaled abundance. Euclidean distance was computed between the rows and row-wise clustering was performed using the complete method. n biological replicates = 3–5. [file Image_3.TIF]

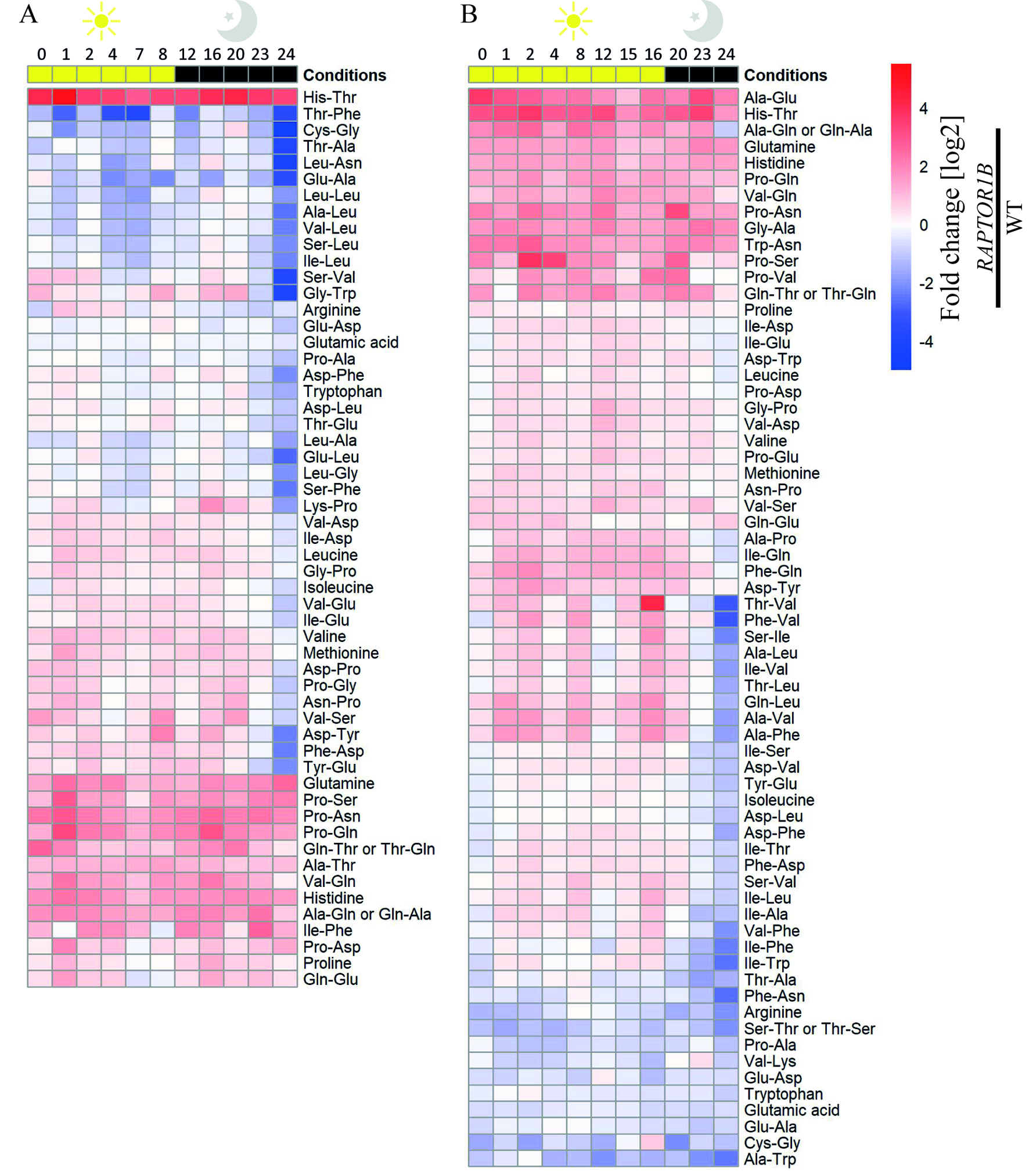

Supplement: Supplementary Figure 4 — Fold change values of the oscillating amino acid and dipeptide levels in A. thaliana raptor1b over Col-0 plants throughout a diel cycle (24 h). Fold change [log2] of the dipeptides and amino acid levels significantly (adjusted P-value ≤ 0.05) affected throughout a diel cycle at (A) SD and (B) LD conditions are illustrated as a heatmap. Euclidean distance was computed between the rows and row-wise clustering was performed using the complete method. [file Image_4.TIF]

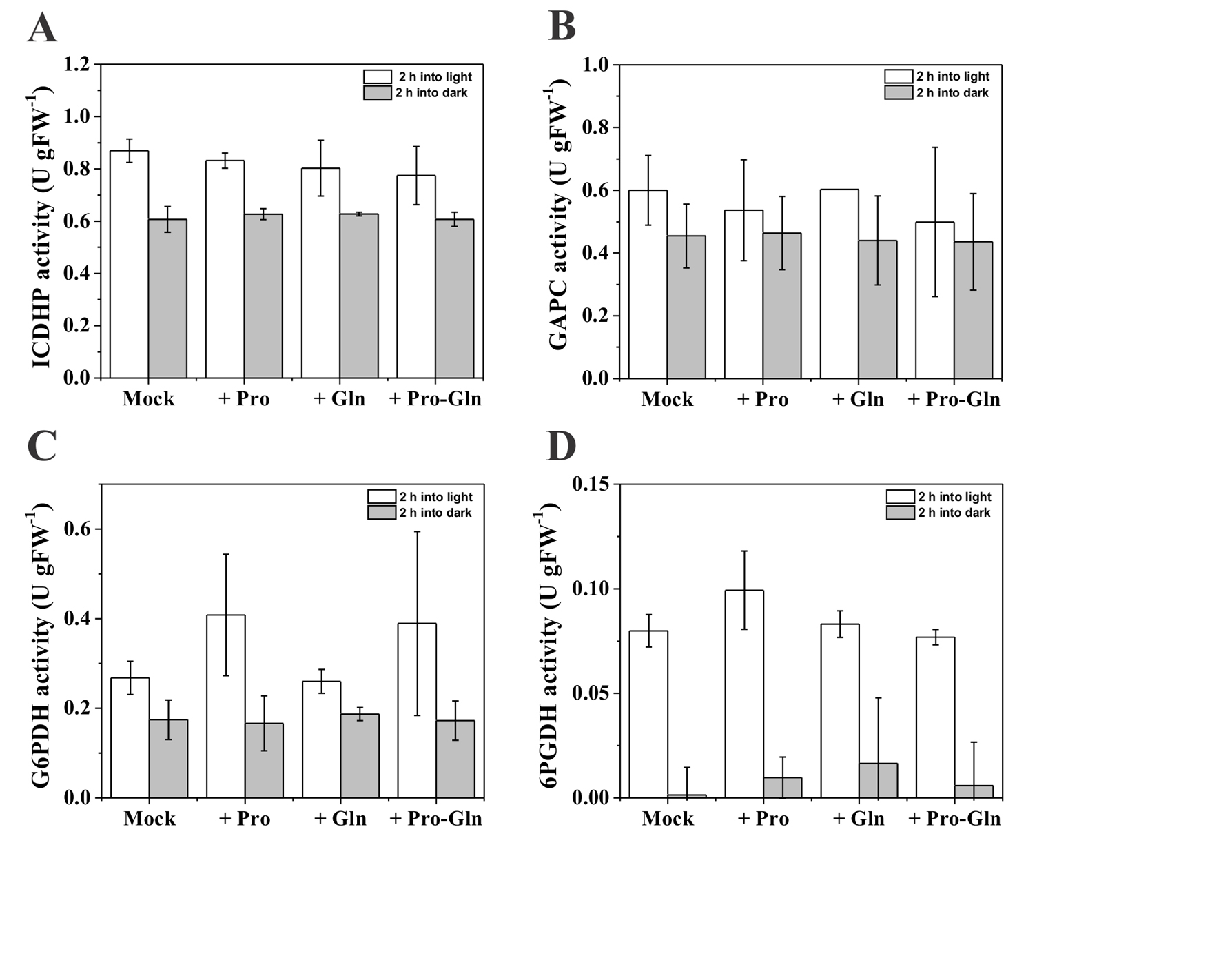

Supplement: Supplementary Figure 5 — Enzymatic activity measurements of Pro-Gln putative protein interactors. (A) ICDHP, (B) GAPC, (C) G6PDH, and (D) 6PGDH activity measurements under control conditions (mock) or with the addition of 100 μM Pro, Gln, or Pro-Gln. Enzymatic assays were performed on plant lysates harvested 2 h into the day (white bars) and 2 h into the dark (gray bars). Data are the mean of at least two independent data sets ± standard error. [file Image_5.JPEG]
